# Pharmacotherapy agents in prevention and treatment of breast cancer-related lymphedema: a systematic scoping review

**DOI:** 10.3389/fonc.2026.1751628

**Published:** 2026-03-04

**Authors:** Caroline Lommer, Lila Schroeder, Caroline Amato, Kerry Dhakal, Caitlin Kotian, Dionisia Quiroga, Electra D. Paskett, Mei R. Fu, Ann Scheck McAlearney, Stephanie Collins, Tari A. King, Sarah A. McLaughlin, Sara P. Myers

**Affiliations:** 1 The Ohio State University College of Medicine, Columbus, OH, United States; 2 Division of Surgical Oncology, Department of Surgery, The Ohio State University Wexner Medical Center and James Cancer Hospital, Columbus, OH, United States; 3 The Center for the Advancement of Team Science, Analytics, and Systems Thinking in Health Services and Implementation Science Research (CATALYST), College of Medicine, The Ohio State University, Columbus, OH, United States; 4 Health Sciences Library, The Ohio State University, Columbus, OH, United States; 5 Oncology Rehabilitation, The James Cancer Hospital and Solove Research Institute, The Ohio State University Wexner Medical Center, Columbus, OH, United States; 6 Division of Medical Oncology, Department of Internal Medicine, The Ohio State University Wexner Medical Center and James Cancer Hospital, Columbus, OH, United States; 7 School of Nursing and Health Studies, University of Missouri-Kansas City, Kansas, MO, United States; 8 Comprehensive Cancer Center, The Ohio State University, Columbus, OH, United States; 9 Department of Family and Community Medicine, College of Medicine, The Ohio State University, Columbus, OH, United States; 10 Division of Breast Surgery, Department of Surgery, Emory University School of Medicine, Atlanta, GA, United States; 11 Winship’s Glenn Family Breast Center, Winship Cancer Institute of Emory University, Atlanta, GA, United States; 12 Division of Surgical Oncology, Mayo Clinic, Jacksonville, FL, United States

**Keywords:** breast, breast cancer related lymphedema (BCRL), lymphedema, pharmacotherapy, upper extremity

## Abstract

**Background:**

Breast cancer-related lymphedema (BCRL) is a common and life-long adverse event affecting ~20% of breast cancer survivors. As existing non-pharmacologic management is burdensome, expensive, and variably effective, this systematic scoping review aims to identify pharmacologic and herbal agents for prevention and treatment for BCRL.

**Methods:**

PubMED, Embase, Web of Science Core collection, and the Cumulative Index to Nursing and Allied Health Literature were searched for studies published in English between 1993 and 2025 that investigated the preventative or therapeutic effect of pharmacologic or herbal agents on BCRL among adult stage I-III breast cancer patients. Studies describing interventions with systemically absorbed anti-inflammatories, anti-thrombotics, anti-coagulants, and blood product components were included. Systematic reviews, protocols for ongoing clinical trials, preclinical and non-human studies, editorials, and studies not exclusive to BCRL were excluded. Three reviewers screened and extracted data between June and August 2025. The primary outcomes of interest were reduction in BCRL incidence or severity.

**Results:**

Of the 217 articles screened, 37 were included in the final review. After full text review, 13 were excluded for repetitive data, non-English language, or irrelevant outcomes. The 24 studies included in the analysis investigated anti-diabetic, herbal, anti-inflammatory, anti-hypertensive, immunomodulatory, and microbiome modifying agents, and venoactive flavinoid derivates. Three studies explored the role of pharmacologic/herbal agents in BCRL prevention. While thiazolidinediones, anti-hypertensives, and non-steroidal anti-inflammatory drugs (NSAIDs) had no effect on BCRL incidence, glucagon-like peptide-1 receptor agonists (GLP-1 RA) were associated with BCRL prevention. In the 21 studies that assessed the effect of pharmacologic/herbal agents in BCRL treatment, NSAIDs/steroids, anti-hypertensives, microbiome/synbiotic supplements, and doxycycline showed no benefit and data for flavonoid-derived venoactive agents and herbal products were inconsistent. Immune-modulating therapies were associated with improved BCRL signs/symptoms in three studies.

**Conclusion:**

This systematic scoping review found limited evidence suggesting that GLP-1 RAs may reduce the risk of BCRL and that immunomodulatory agents may improve signs/symptoms of BCRL. Rigorous prospective trials using standardized limb volume/edema, quality-of-life (QoL), and symptom measures and longer follow-up are needed to inform clinical practice aimed at preventing and treating BCRL.

**Systematic review registration:**

https://www.crd.york.ac.uk/PROSPERO/, identifier CRD420251055134.

## Introduction

Breast cancer-related lymphedema (BCRL) is a common, potentially debilitating complication affecting about 20% of breast cancer (BC) survivors (1). As symptom severity does not necessarily correlate with disease severity (2), patients with subclinical or low-stage BCRL may still experience substantial functional limitations, swelling of and pain in the breast, chest wall, or upper extremity, as well as psychological distress (3). Sequelae of impaired lymphatic flow, such as recurrent infections and cellulitis, can exacerbate these issues (4, 5). In addition, costs associated with BCRL treatment and vocational disruption from permanent disability are associated with long-term financial hardship (6).

Despite advancements in care, BCRL management remains a significant challenge for patients and providers. First-line treatment involves life-long compression and/or complete decongestive therapy (CDT), with surgical interventions reserved for refractory and advanced-stage BCRL (7). Variable efficacy (8) and limited insurance coverage (9) have motivated prioritization of prevention and risk reduction approaches. Although existing data indicate that prospective surveillance models with early intervention may reduce BCRL incidence and severity (10), implementation barriers hamper widespread adoption (11). Surgical procedures aimed at prevention, such as immediate lymphatic reconstruction (12, 13), require surgeons trained in microvascular techniques, have limited long-term follow-up data, and are infrequently reimbursed by insurance (14).

An increasing body of literature is exploring pharmacologic and herbal agents as alternatives for prevention and treatment of secondary lymphedema that might circumvent the aforementioned challenges. Examples include pharmacotherapies such as soluble TNF-α receptor 1 inhibitors, ketoprofen, tacrolimus, and cyclophosphamide that reduce tissue inflammation and fibrosis due to lymphedema (15). While some studies have observed benefit, variations in study design, patient population, and outcome measures limit generalizability and clinical application (16). As a first step in designing future investigations to support the development of effective pharmacologic strategies for both BCRL prevention and treatment, we conducted a systematic scoping review to synthesize data on the efficacy of pharmacotherapeutic agents and highlight gaps in knowledge.

## Methods

### Study design

This systematic scoping review aimed to evaluate the efficacy of systemically absorbed pharmacotherapy agents and herbal supplements in preventing or treating BCRL among adult patients ≥ 18 years old at the time of diagnosis with stage I-III BC who either developed BCRL or were at risk for BCRL following BC treatment. Eligible comparators included placebo, standard of care (e.g., compression therapy, CDT), alternative pharmacologic agents, or no treatment. In studies without an explicit comparator arm, pre-post changes were evaluated descriptively in alignment with scoping review methodology. Primary outcomes, as elaborated below, included incidence of BCRL for prevention studies or change in BCRL severity in response to treatment. This study was conducted according to the JBI Manual for Evidence Synthesis (17) and adheres to the Preferred Reporting Items for Systematic Reviews and Meta-Analyses (PRISMA) guidelines (18). The review protocol is registered with PROSPERO, registration ID CRD420251055134 (19).

### Data sources and search strategy

PubMED, Embase (Elsevier), Web of Science Core Collection (Clarivate), the Cumulative Index to Nursing and Allied Health Literature (CINAHL, EBSCO) were searched for studies that investigated the effects of anti-inflammatories, anti-thrombotic agents, anti-coagulants, and blood product components on BCRL severity (**Supplementary Table S2**). Surgical, device-based, and rehabilitative interventions were outside the prespecified scope of this review. A medical librarian (KD) and two reviewers (SPM, DQ) designed the search strategy, which queried Medical Subject Heading (MeSH) and entry terms for non-steroidal anti-inflammatory agents (NSAIDs) (aspirin, ketoprofen, ibuprofen), anti-coagulants (apixaban, rivaraxaban, edoxaban, fondaparinux, heparin, dalteparin, enoxaparin, argatroban, bivalirudin, dabigatran, desirudin, warfarin), cyclosporine, hydroxychloroquine, tacrolimus, sirolimus, leukotriene antagonists, platelet-rich plasma, immunosuppressive agents, herbs/medicinal plants, and dietary supplements. The search was carried out on May 24^th^, 2025. In addition to results generated from the final search strategy, a *post hoc* hand search was performed to identify relevant studies not captured by indexing or database coverage. This approach was intended to identify emerging therapies and novel pharmacological agents that may have been recently published or not indexed. The complete list of generic and brand-name agents is provided in **Supplementary Table S1**.

### Study selection and eligibility

Peer-reviewed studies published in English between 1993 and 2025 that reported the primary outcomes of interest, incidence or change in severity of BCRL, were included. Eligibility criteria for study inclusion were if the studies (1) focused on adult patients diagnosed with stage I-III BC; (2) used systemically absorbed pharmacologic agents to prevent or treat BCRL; (3) used either quantitative or qualitative measures to understand change in severity; (4) used quantitative measures of limb volume, arm circumference, bioimpedance score (BIS), and clinical staging. Studies that considered topical agents or preclinical investigations were excluded. Data pertaining to quality-of-life (QoL), though not considered a primary outcome of interest, was collected and is available in the **Supplementary Material**. Qualitative assessments included patient-reported outcomes addressing arm heaviness, hardness, perceived disability, tightness/tension, and pain. Systematic reviews, protocols for ongoing clinical trials, preclinical and non-human studies, editorials, conference abstracts, and studies not exclusive to patients with BCRL were excluded. Although surgical, device-based, topical, and rehabilitative programming interventions were excluded *a priori*, studies were included if these interventions were delivered concomitantly with a pharmacologic or herbal agent and the latter was the exposure of interest.

### Data extraction, processing, and synthesis

Three reviewers (LJS, CL, CA) independently screened and reviewed articles and abstracted data related to primary and secondary outcomes. Articles were screened using Covidence systematic review software (Veritas Health Innovation, Melbourne, Australia) (20). Two reviewers (CL and SPM) created a data abstraction form. Duplicate records, titles, and abstracts were removed. An additional reviewer (DQ) resolved disagreements or discrepancies.

Given the substantial heterogeneity across included studies, a meta-analysis was not feasible. Instead, we conducted a structured narrative synthesis following JBI and PRISMA-ScR guidance (17, 18). Study quality and risk of bias were assessed using the National Heart, Lung, and Blood Institute Quality Assessment Tools (21), selected by study type. While no formal weighting was applied, the risk of bias assessments informed interpretation of findings.

## Results

### Study selection and characteristics

**Figure 1:**
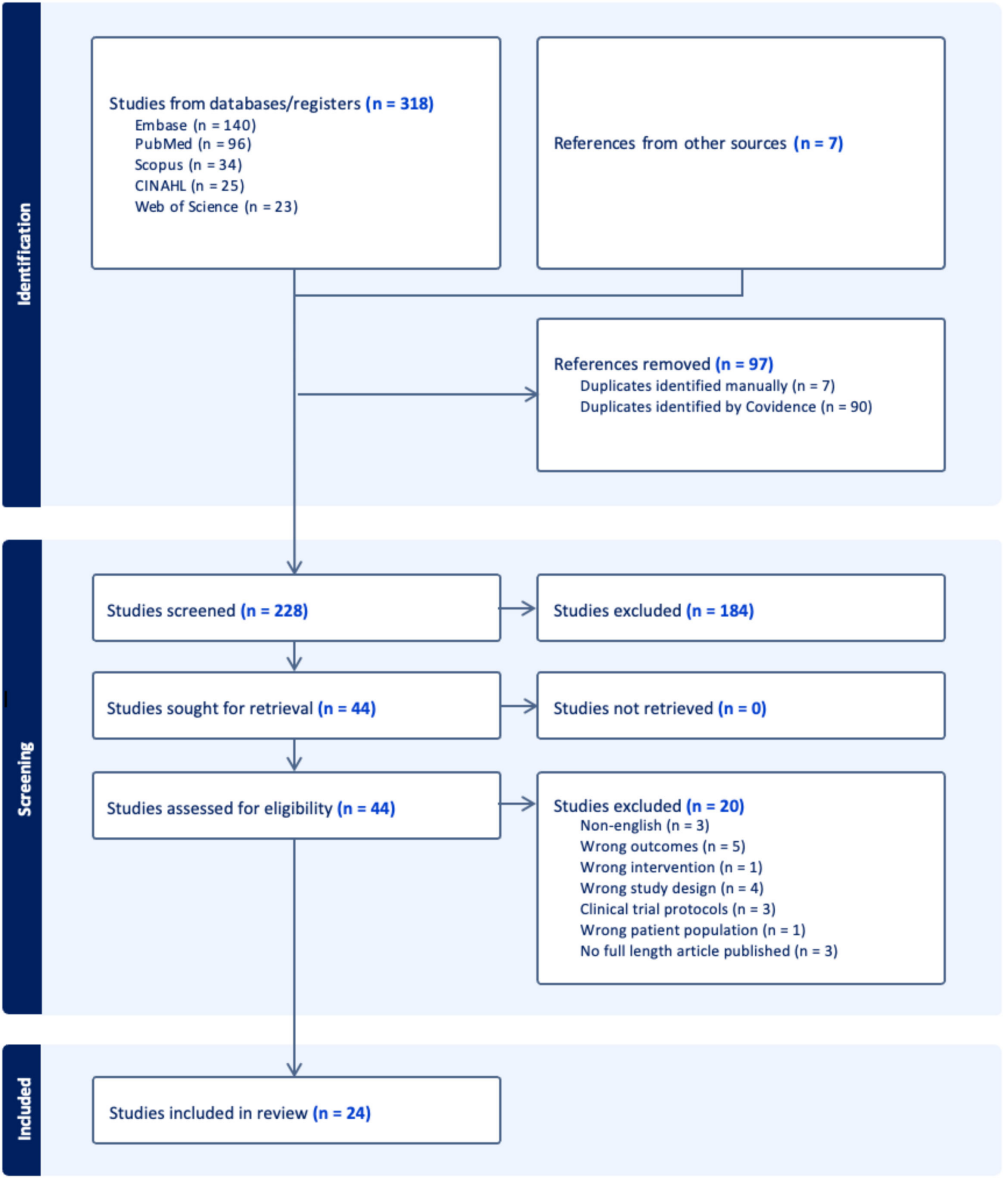
PRISMA flow diagram of the systematic review process (18). A total of 318 records were identified through database searches and 7 from other sources (hand picked). After removing 97 duplicates, 228 records were screened. Of these, 184 were excluded, and 44 full-text articles were assessed for eligibility. Twenty articles were excluded. Ultimately, 24 studies were included in the review.

**Figure 2:**
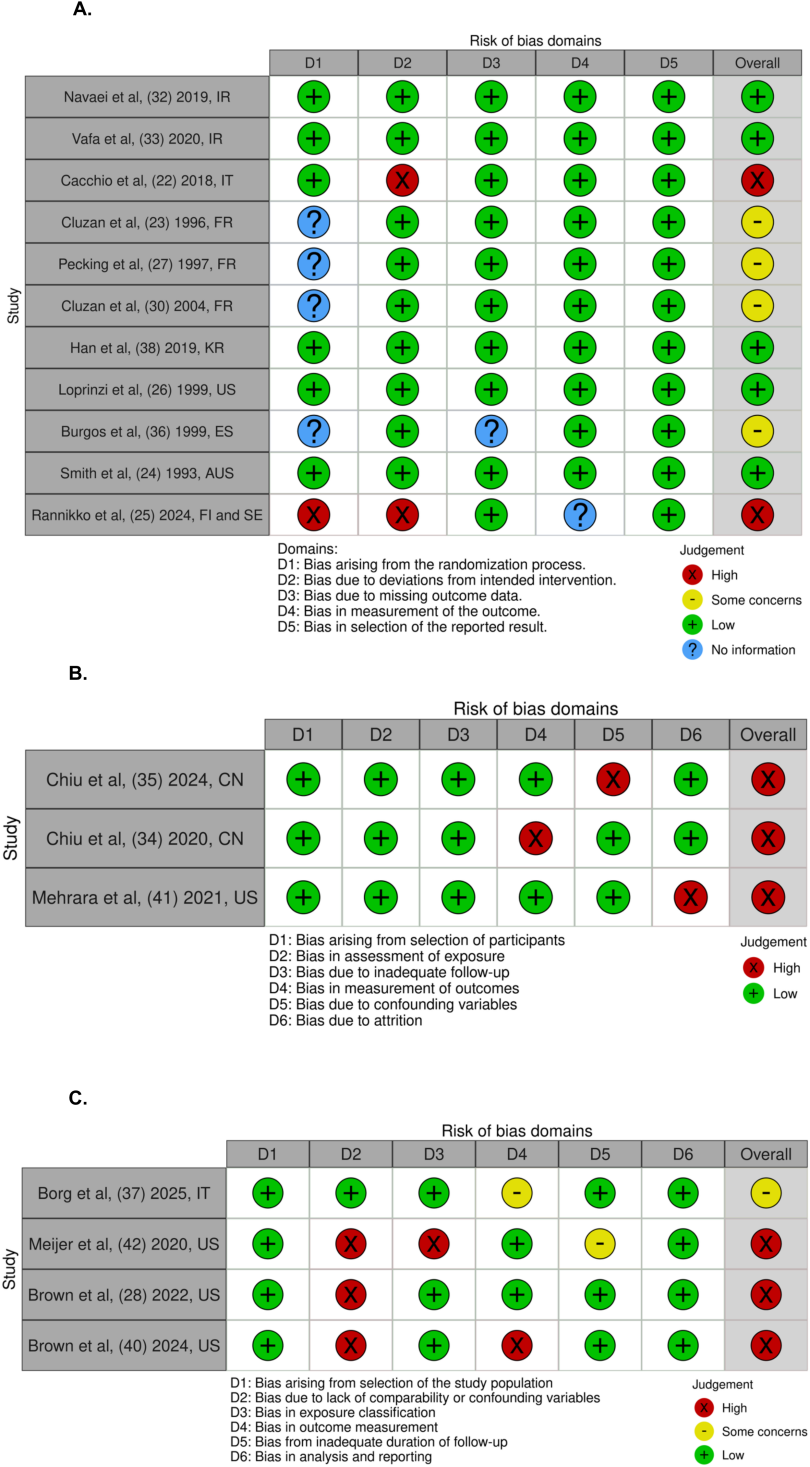
**(A–C)** present graphical summaries of the risk of bias assessments for all included studies using a traffic-light color-coding scheme. **(A)** Summarizes randomized controlled trials (RCTs), **(B)** summarizes prospective cohort studies, and **(C)** summarizes retrospective cohort studies. Green indicates low risk of bias, yellow indicates some concerns, red indicates high risk of bias, and blue indicates insufficient information. Randomized controlled trials (RCTs) were evaluated using domains from the Cochrane Risk of Bias tool. Retrospective and prospective cohort studies were assessed with criteria adapted from the Newcastle–Ottawa Scale and NIH Quality Assessment Tools. **(A)** Randomized Controlled Trials. **(B)** Prospective Cohorts. **(C)** Retrospective Cohorts.

The search resulted in 217 unique records (**Figure 1**). Of these, 180 were excluded due to irrelevant content in the title and abstract. Thirty-seven articles were included in the full-text review. A total of 24 studies, the majority of which were randomized controlled trials (n = 13) were included (**Tables 1A, B**). Twenty-one studies focused on pharmacotherapy for BCRL treatment (1B), whereas 3 evaluated agents in a preventative context (1A). To characterize treatment response, studies used several different methods to quantify changes in BCRL severity. Studies described four primary approaches to quantify improvements in BCRL severity: 1) serial circumferential measurements to estimate limb volume indirectly (25, 28–30, 32, 34, 38–40), 2) displaced water volume techniques to measure changes in limb volume (22, 26, 27, 33, 35, 36, 41), and 3) bioelectrical impedance analysis to estimate limb fluid distribution and body composition (23, 31, 42) 4) perometry utilizing infrared light to create a three-dimensional image of the affected limb (24, 37). Definitions and staging of BCRL were not standardized across the 24 studies. Several studies adhered to International Society of Lymphology (ISL) guidelines (23, 25, 31, 33, 34, 37, 40) while others applied internal diagnostic criteria or relied on absolute limb circumference differences without established validation (22, 24, 26–30, 32, 35, 36, 38, 39, 41, 42). Duration of therapies tested ranged from two weeks to 12 months. Co-interventions, which were inconsistently reported, included calorie restriction and CDT or components of CDT- compression garments, manual lymphatic drainage, or other exercise-based interventions. Only three studies conducted follow-up assessments after treatment cessation (25, 31, 37). The heterogeneity of the studies in pharmacologic and herbal agents as well as outcome measures limited this review to conduct further meta-analysis. Risk of bias assessment highlighted other challenges to data synthesis and interpretation including clearly defined study population, research objectives, and interventions (**Figures 2A–C**). Overall, randomized controlled trials were generally of moderate quality (2A); prospective cohorts were largely at low risk of bias (2B); and retrospective cohorts demonstrated variable but overall low-to-moderate quality (2C).

**Table 1:** Summary of included clinical studies evaluating pharmacotherapeutic agents for the prevention (1A) or treatment (1B) of BCRL.

1A. Clinical studies evaluating agents used in prevention of BCRL				
Study	Design	Drug, dose, duration	Primary outcome	Findings
Borg et al (22), 2025, IT	Retrospective cohort (N = 162)	Anti-hypertensives, NSAIDS, steroids, anti-diabetics	Physician clinical diagnosis of BCRL	No significant differences
Brown et al (23), 2024, US	Retrospective cohort (N = 3830)	GLP-1 RAs	Incidence of Lymphedema	OR 0.14, 95% CI 0.04-0.32, *p* < 0.0001
Meijer et al (24), 2020, US	Retrospective case-control (N = 345)	Aldosterone synthase inhibitors, calcium channel blockers, NSAIDs	Percentage increase in arm volume	No significant differences
1B. Clinical studies evaluating agents used in treatment of BCRL				
Study	Design	Drug, dose, duration	Primary outcome	Findings
Cacchio et al (25), 2018, IT	RCT (N = 50)	234.3 mg Lifadren x 6 weeks	Excess limb volume	73.6% vs 31.3% reduction (p < 0.0001)
Chiu et al (26), 2024, CN	Prospective cohort (N = 20)	30 g Peoniae rubra and Astragulus x 6 months	Limb volume of the affected arm	No significant difference
Chiu et al (27), 2020, CN	Prospective cohort (N = 9)	30 g Peoniae rubra and Astragulus x 6 months	Limb volume of the affected arm	No significant difference
Cluzan et al (28), 1996, FR	RCT (N = 57)	300 mg Cyclo-3-Fort x 3 months	Edema volume	12.9% reduction vs 2.55% increase (p = 0.009)
Pecking et al (29), 1997, FR	RCT (N = 94)	500 mg Daflon x 6 months	Edema volume	No significant difference
Cluzan et al (30), 2004, FR	RCT (N = 48)	600 mg BN165 (Ginkor Fort) x 2 months	Swelling	No significant difference
Han et al (31), 2019, KR	RCT (N = 26)	500 ug Sodium Selenite x 2 weeks	ISL clinical stage of lymphedema	83.3% vs. 10.0% stage change from III to II (p = 0.002)
Loprinzi et al (32), 1999, US	RCT – crossover (N = 93)	200 mg Coumarin x 6 months	Excess limb volume	58 mL vs 21 mL increase (p=0.80).
Burgos et al (33), 1999, ES	RCT (N = 53)	105 mg Lysedem x 12 months	Volume of lymphedema	No significant difference
SMIth et al (34), 1993, AUS	RCT – crossover (N = 52)	400 mg Coumarin x 6 months	Excess limb volume	46% vs 26% reduction (p < 0.001)
Navaei et al (35), 2019, IR	RCT (N = 80)	Lactocare x 10 weeks	Excess limb volume	No significant difference
Vafa et al (36), 2020, IR	RCT (N = 121)	Lactocare x 10 weeks	Edema volume	No significant difference
Mehrara et al (37), 2021, US	Prospective cohort (N = 9)	QBX258 x 4 months	Excess limb volume	No significant difference
Brown et al (38), 2022, US	Retrospective cohort (N = 17)	Doxycycline	Limb volume of the affected arm	No significant difference
Leppäpuska et al (39), 2020, FI	Prospective cohort (N = 27)	Lymfactin x 24 months	Excess limb volume	No significant difference
Rannikko et al (40), 2024, FI and SE	RCT (N = 39)	Lymfactin x 12 months	Relative volume difference ratio	No significant difference
Belcaro et al (41), 2018, IT	Registry Study (Non-Randomized, Open-Label) (N = 65)	Robuvit x 2 months	Limb volume reduction	-19.82% vs. -12.81% (p < 0.05)
Pereira de Godoy et al (42), 2018, BR	Prospective cohort (N = 13)	Daflon x 1 month	Unclear	Significant reduction in limb volume p < 0.04

The table presents randomized controlled trials (RCTs), prospective cohorts, retrospective cohorts, and registry studies investigating various agents. Study characteristics, drug and dose duration, primary outcome, and main findings are listed.

### Anti-diabetic medications

Two studies examined anti-diabetic medications (22, 23). GLP-1 receptor agonists (GLP-1 RAs) may reduce BCRL risk by improving insulin sensitivity, decreasing pro-inflammatory signaling, and preserving lymphatic vessel integrity. Brown et al. (23) reported that patients (n = 36) receiving GLP-1 RAs after axillary lymph node dissection had significantly lower odds of developing BCRL at a median 75-month follow-up (OR 0.16, 95% CI 0.05–0.40; p < 0.0001), with minor adverse events reported (nausea, diarrhea, vomiting, and constipation). The usage international classification of disease codes was used to assess those who developed lymphedema. Conversely, risk for developing BCRL in patients taking thiazolidinediones, which can cause fluid retention, were evaluated in a retrospective cohort of 162 post-surgical survivors (22). After adjustment for confounders, thiazolidinediones were not associated with increased BCRL risk, assessed via circumferential measurement.

### Herbal therapies

Four studies evaluated herbal agents (26, 27, 31, 41). In their randomized controlled trial (RCT), Han et al. (31) found that five sessions of intravenous sodium selenite infusion over two weeks compared to regular saline infusion, assessed by BIS, was associated with clinical improvement from stage III to stage II BCRL (10/12 (83%) selenium vs 1/11 (9%) placebo, p = 0.002) at one month post-intervention. Two publications assessed the herbal formulation of *Paeoniae rubra* (moutan cortex) and *Astragulus*, which has been shown to promote lymphatic flow and reduce interstitial fluid accumulation (26, 27). Both studies utilized water displacement for limb volume and tape measurement for limb circumference. One of these studies represented an expanded prospective cohort building on the other, and neither reported statistically significant improvement in arm volume, measured via water displacement, after 6 months of supplementation (p > 0.05–2020 study; p = 0.068 in 2024). Belcaro et al. (41) conducted a non-randomized registry study comparing arm volume in 32 patients who received Robuvit, a natural French oak wood extract proposed to reduce inflammation, as an adjunct to CDT with 33 patients who received CDT alone. After two months, mean volume reduction was greater in the Robuvit^®^ plus CDT group (-654 ± 21 mL) (p < 0.05) than in the CDT-only group (-433 ± 23 mL).

### Anti-inflammatory agents and steroids

Two retrospective cohort studies considered anti-inflammatory and steroidal agents (22, 24). In their retrospective cohort of 162 post-surgical BC survivors, Borg et al. (22) did not observe a significant association between NSAIDs (prescribed to 6 patients) or corticosteroids (prescribed to 8 patients) and BCRL development. Similarly, no significant associations were noted in the retrospective case-control study performed by Meijer et al. (24) between NSAIDs (p = 0.971), corticosteroids (p = 0.999) or aspirin (p = 0.449) and BCRL development. Limb volume was measured via perometry and utilized relative volume change formula.

### Anti-hypertensives

Two previously mentioned studies also included anti-hypertensives (22, 24), which have been postulated to lead to fluid retention and peripheral edema given increased capillary hydrostatic pressure.

In the Borg et al. (22) study, calcium channel blockers, minoxidil, methyldopa, hydralazine, clonidine, and beta blockers, were prescribed to 49 patients, of whom 35 subsequently developed BCRL. On multivariable analysis, none of these agents were significantly associated with increased risk for BCRL. Meijer et al. (24) also investigated the effect of calcium channel blockers (amlodipine, nifedipine, felodipine, diltiazem) and similarly observed no significant association between these agents and BCRL.

### Flavonoid-derived venoactive agents

Eight studies explored flavonoid-derived venoactive compounds, including diosmin and coumarin (25, 28–30, 32–34, 42). Results were inconsistent. In their crossover RCT, Loprinzi et al. (32) found no significant difference in arm volume between placebo and 6 months of oral coumarin. They utilized tape measurements and calculated volume using the formula for the volume of a cylinder. No difference in arm volume, measured via the opto-electricon device (Volumeter^®^), was noted in the Burgos et al. (33) multi-center RCT comparing two doses of Lysedem (coumarin and troxerutin). Pecking et al. (29) conducted an RCT of Daflon (diosmin and hesperidin) versus placebo with no observed difference in limb volume via perometry. Cluzan et al. (30) evaluated two different doses of BN165 (coumarin, proanthocyanidins and flavones) to placebo in an RCT; after two months, there was no statistically significant difference in arm swelling, as assessed by perometer.

In contrast, several studies did find benefit. Smith et al. (34) conducted a 12-month crossover RCT of coumarin versus placebo in patients with stage II BCRL and noted that limb volume decreased from 46% to 26% above the unaffected limb (p < 0.001). Limb volume was measured by water-displacement. In their RCT, Cacchio et al. (25) compared Linfadren (diosmin, coumarin, arbutin) combined with CDT with CDT alone in 48 patients. After six weeks of treatment, measured by tape measure using the truncated cone method, mean edema volume decreased by 521 mL (from 581.8 to 460.1 mL) in patients receiving Linfadren plus CDT, compared to a 265 mL reduction (from 330.1 to 199.8 mL) with CDT alone (p <.0001). Three months post-treatment, mean limb volume in both groups remained stable, and no adverse events were reported. Cluzan et al. (28) found that after 3 months of treatment with Cyclo-3-Fort (ruscus aculeatus, hesperidin methyl chalcone, and ascorbic acid), there was a 12.9% decrease in upper limb volume compared to a 2.6% increase in the placebo group (p = 0.009). Limb volume was determined using the truncated cone method. Pereira de Godoy et al. (42) prospectively evaluated Daflon in patients with mild BCRL for one month. BIS analysis showed improvement in limb fluid content (p < 0.04).

### Immune modulating and novel biologic agents

Three studies investigated agents that could increase circulating VEGF in the prevention (1) (40) or treatment (2) (38, 39) of BCRL. Rannikko et al. (40) evaluated Lymfactin^®^, a VEGF-C gene therapy designed to promote lymphangiogenesis and restore lymphatic flow, in patients undergoing vascularized lymph node transfer. After 12 months, excess arm volume decreased by a median of 30.0% (136 ± 189 mL) in the treatment group compared with 23% (164 ± 133 mL) in the placebo group (p > 0.05). Arm volume was measured by tape measure using the truncated cone method. A phase I trial by Leppäpuska et al. (39) treated patients with two doses of Lymfactin^®^ and found that the higher-dose group demonstrated an average 46% reduction in excess arm volume at 12 months. This study also measured arm volume by tape measure using the truncated cone method. A retrospective cohort from Brown et al. (38) assessed the impact of 6 weeks of oral doxycycline, which inhibits VEGF-C signaling, but no changes in disease stage were observed, and differences in limb volume, via truncated cone method and BIS, at 17-week follow-up were not statistically significant.

One study explored immune modulation through cytokine blockade. Mehrara et al. (37) administered QBX258, a monoclonal IL-4/IL-13 neutralizing antibody, to nine patients with stage I–II BCRL for four months with 16–20 weeks of follow-up. Volume differences assessed using Perometry showed clinical improvements, although detailed quantitative outcomes were limited.

### Microbiome-modifying agents

Two studies investigated synbiotic supplements, which combine prebiotics and probiotics and are hypothesized to improve intestinal homeostasis and attenuate systemic inflammation by downregulating pro-inflammatory cytokines (35, 36). Navaei et al. (35) performed a trial of patients with stage I–II BCRL randomized to receiving Lactocare plus a low-calorie diet or a low-calorie diet alone for 10 weeks. Excess limb volume, measured using water displacement, showed no significant difference between the groups, and no additional co-interventions were included. A subsequent trial by Vafa et al. (36) evaluated a similar synbiotic formulation. This trial enrolled 121 patients with stage I–II BCRL randomized to a synbiotic plus calorie restriction, placebo plus calorie restriction, or placebo alone. CDT was permitted as a co-intervention. After 10 weeks, edema volume, measured via water displacement, decreased significantly in the calorie-restricted synbiotic group compared to placebo (p = 0.002); however, the difference between the calorie-restricted synbiotic and calorie-restricted placebo groups was not significant. The observed reduction in edema volume was attributed primarily to calorie restriction rather than synbiotic supplementation.

## Discussion

This systematic review identified 24 studies evaluating pharmacotherapeutic and herbal agents for BCRL, the majority of which addressed treatment rather than prevention. Of the studies that investigated agents for prevention, GLP-1 RAs were protective, albeit data was restricted to a single study. With respect to agents used to treat BCRL, NSAIDs/steroids, anti-hypertensives, synbiotics, and doxycycline showed no consistent benefit, flavonoid-derived venoactive and herbal agents produced mixed results, and immunomodulatory strategies showed trends of improvement.

The observed association of GLP-1 RAs in prevention aligns with the known systemic metabolic effects and theorized anti-inflammatory pathways for these agents. Interestingly, weight loss which has previously been examined as a therapeutic rather than preventative strategy for lymphedema (43), has yielded inconsistent results, suggesting that the protective mechanisms of GLP-1 RAs may involve complex pathways beyond simple weight reduction. Systemically, GLP-1 RAs modulate adipokine signaling by increasing anti-inflammatory adiponectin and reducing leptin. In this way, GLP-1 RAs counteract the pro-inflammatory cytokine release (TNF-α, IL-6) and fibroadipose tissue deposition stimulated by leptin (44). Importantly, GLP-1 RAs are also being investigated for potential survival benefits in breast cancer, with several ongoing trials exploring their impact on cancer outcomes (45–47). In investigating agents that may be used for prevention, our review also identified medications that may increase risk for developing BCRL; specifically, one publication noted a potential association between thiazolidinediones (22), an anti-diabetic agent known to cause fluid retention (48), and BCRL development. Yet, paradoxically, rosiglitazone, a pro-adipogenic thiazolidinedione that functions as a peroxisome proliferator activated receptor gamma (PPARγ) agonist, reduced tissue fibrosis in a mouse model of secondary lymphedema after hindlimb lymphadenectomy (48). These findings highlight the complex relationship between metabolic regulation and lymphatic function and underscore the need for further research on anti-diabetic medications, including GLP-1 receptor agonists and thiazolidinediones, to clarify their potential therapeutic and adverse effects in BCRL.

Chronic inflammation and immune dysregulation are central to lymphedema pathophysiology, driving tissue fibrosis, adipose deposition, and impaired lymphatic repair. While this review included retrospective studies evaluating NSAID use in relation to BCRL development (22, 24), specifically, no trials have specifically investigated NSAIDs as a therapeutic intervention for established BCRL. In an open-label clinical trial (n = 21), ketoprofen reduced skin thickness, improved composite histopathology scores, and decreased plasma granulocyte colony-stimulating factor levels in patients with primary or secondary lymphedema (49). Similarly, animal models demonstrated that ketoprofen attenuated TNF-α signaling and improved lymphatic vascular structure in secondary lymphedema (15). Other approaches have aimed to stimulate lymphangiogenesis directly; for example, stromal vascular fraction therapy (containing stem cells, immune cells, and regenerative cell types) increased VEGF-C expression and reduced edema in a rabbit hindlimb model (50). Translating VEGF-C stimulation into clinical therapy, however, remains challenging because VEGF inhibition has been used in cancer treatment, raising concerns about tumor growth and recurrence risk. More broadly, this extends to many of the agents identified in our review, as it remains unknown whether these pharmacologic or biologic interventions may affect BC recurrence or interfere with cancer therapy. Additionally, herbal supplements investigated for their potential effects on lymphedema are not regulated by the Food and Drug Administration. Thus, it remains unclear whether certain compounds could carry estrogenic, pro-angiogenic, or pro-mitogenic potential, thereby posing additional risks to BC survivors. In contrast, doxycycline, studied primarily in filariasis-associated lymphedema, reduced disease severity through immunomodulation, including inhibition of monocyte recruitment and suppression of adaptive T cell responses, which coincided with lower circulating VEGF-C and soluble VEGFR-3 levels (51–53]. Collectively, these varied therapeutic targets highlight the complexity of inflammatory signaling in lymphedema and suggest that treatment effects may depend on timing, underlying etiology, and disease stage. Mechanistic complexity and heterogeneity of BCRL patient populations likely contribute to the limited and inconsistent clinical evidence supporting pharmacologic interventions. Future studies are needed not only to assess the efficacy of these interventions in BCRL but also to clarify their impact on tumor recurrence and survival outcomes.

This systematic scoping review has strengths and limitations worth noting. While prior reviews have focused primarily on conservative, rehabilitative, or surgical management strategies for BCRL, to our knowledge none have systematically evaluated systemically absorbed pharmacologic or herbal agents for prevention and/or treatment of lymphedema specifically due to breast cancer. Although this pharmacotherapy-centered perspective offers new insights into disease-modifying mechanisms and potential preventative strategies, several limitations affect interpretation of data. The majority of studies included small, single-center trials or retrospective cohorts, limiting generalizability and increasing susceptibility to bias. Randomization and blinding were inconsistently reported, and selective outcome reporting was common. The heterogeneity of outcome measures, co-interventions (e.g., compression therapy, calorie restriction), and diagnostic criteria precluded meta-analysis and limited synthesis of quantitative effect estimates. Although certain pharmacologic subgroups initially appeared suitable for exploratory meta-analysis, closer examination of this substantial heterogeneity in outcome definitions, measurement methods, treatment regimens, and study design would have rendered any pooled estimate unreliable or potentially misleading. Differences in treatment duration, intervention dosage, co-intervention strategies, and follow-up timelines likely account for inconsistencies in studies’ findings. Measurement techniques varied across study designs as some studies measured limb circumference, utilized water displacement, relied on an opto-electronic device, or used both water displacement and limb circumference. Variation in QoL assessment and use of validated measures also restricted data quality and generalizability. By design, our search did not include studies evaluating surgical, microsurgical, or device-based interventions for BCRL. Investigations highlighting these prevention/treatment strategies are available and have contributed significantly to the treatment of BCRL (51, 52). While relatively few systemic pharmacologic agents demonstrated therapeutic benefit, this reflects the current paucity of rigorous trials and underscores the need for continued investigation into systemic therapies as an adjunct to, rather than a replacement for, established multimodal BCRL management, which includes CDT and surgical approaches addressed extensively in other reviews.

## Conclusion

In this systematic review of 24 manuscripts investigating the effect of pharmacotherapies on BCRL prevention and treatment, we found promising data, albeit limited, to suggest that GLP-1 RAs and immunomodulatory agents may have an impact on reducing incidence and severity of BCRL. Future trials would benefit from clear edema volume and QoL measurement protocols and long-term follow-up to inform clinical practice aimed at reducing this common treatment-related morbidity.

## Data Availability

The raw data supporting the conclusions of this article will be made available by the authors, without undue reservation.
