# Supplementary material for: Pharmacotherapy agents in prevention and treatment of breast cancer-related lymphedema: a systematic scoping review
Source: Front Oncol. 2026 Mar 4;16:1751628. doi: 10.3389/fonc.2026.1751628 (PMC12995638; doi:10.3389/fonc.2026.1751628)
Supplement: Supplementary file 1 [file DataSheet1.pdf]

53. Furlong-Silva J, Cross SD, Marriott AE, Pionnier N, Archer J, Steven A, Merker SS, Mack M, Hong Y-K, Taylor MJ, et al. Tetracyclines improve experimental lymphatic filariasis pathology by disrupting interleukin-4 receptor–mediated lymphangiogenesis. *J Clin Invest* 131:e140853. doi: 10.1172/JCI140853

54. Donahue PMC, MacKenzie A, Filipovic A, Koelmeyer L. Advances in the prevention and treatment of breast cancer-related lymphedema. *Breast Cancer Res Treat* (2023) 200:1–14. doi: 10.1007/s10549-023-06947-7

55. Hasenoehrl T, Palma S, Ramazanova D, Kölbl H, Dorner TE, Keilani M, Crevenna R. Resistance exercise and breast cancer–related lymphedema—a systematic review update and meta-analysis. *Support Care Cancer* (2020) 28:3593–3603. doi: 10.1007/s00520-020-05521-x

56. Badger C, Preston N, Seers K, Mortimer P. Benzo-pyrones for reducing and controlling lymphoedema of the limbs. *Cochrane Database Syst Rev* (2004) 2004:CD003140. doi: 10.1002/14651858.CD003140.pub2

57. Sheikhi-Mobarakeh Z, Yarmohammadi H, Mokhatri-Hesari P, Fahimi S, Montazeri A, Heydarirad G. Herbs as old potential treatments for lymphedema management: A systematic review. *Complement Ther Med* (2020) 55:102615. doi: 10.1016/j.ctim.2020.102615

58. Hnátek L. [Therapeutic potential of micronized purified flavonoid fraction (MPFF) of diosmin and hesperidin in treatment chronic venous disorder]. *Vnitr Lek* (2015) 61:807–814.

59. Ramelet AA. Clinical benefits of Daflon 500 mg in the most severe stages of chronic venous insufficiency. *Angiology* (2001) 52 Suppl 1:S49-56. doi: 10.1177/0003319701052001S07

60. Bihari I, Guex J-J, Jawien A, Szolnoky G. Clinical Perspectives and Management of Edema in Chronic Venous Disease—What about Ruscus? *Medicines* (2022) 9:41. doi: 10.3390/medicines9080041

61. Xing C, Xiang D, Caiying L. Effects of troxerutin on vascular inflammatory mediators and expression of microRNA-146a/NF-κB signaling pathway in aorta of healthy and diabetic rats. *Korean J Physiol Pharmacol Off J Korean Physiol Soc Korean Soc Pharmacol* (2020) 24:395–402. doi: 10.4196/kjpp.2020.24.5.395

62. Kakkos SK, Nicolaides AN. Efficacy of micronized purified flavonoid fraction (Daflon®) on improving individual symptoms, signs and quality of life in patients with chronic venous disease: a systematic review and meta-analysis of randomized double-blind placebo-controlled trials. *Int Angiol J Int Union Angiol* (2018) 37:143–154. doi: 10.23736/S0392-9590.18.03975-5

63. Gloviczki ML, Kakkos SK, Urbanek T, Chuback J, Nicolaides A. The role of venoactive compounds in the treatment of chronic venous disease. *J Vasc Surg Venous Lymphat Disord* (2025) 13: doi: 10.1016/j.jvsv.2025.102258

64. Chong NJ, Aziz Z. A Systematic Review of the Efficacy of Centella asiatica for Improvement of the Signs and Symptoms of Chronic Venous Insufficiency. *Evid-Based Complement Altern Med ECAM* (2013) 2013:627182. doi: 10.1155/2013/627182

65. Farasati Far B, Behzad G, Khalili H. Achillea millefolium: Mechanism of action, pharmacokinetic, clinical drug-drug interactions and tolerability. *Heliyon* (2023) 9:e22841. doi: 10.1016/j.heliyon.2023.e22841

66. Cheon H, Kim B, Jeon JY. Grape seed proanthocyanidins improve lymphatic drainage and blood perfusion in secondary lymphedema models. *Front Oncol* (2025) 15:1553090. doi: 10.3389/fonc.2025.1553090

67. Fukada K, Kajiya-Sawane M, Matsumoto Y, Hasegawa T, Fukaya Y, Kajiya K. Antiedema effects of Siberian ginseng in humans and its molecular mechanism of lymphatic vascular function in vitro. *Nutr Res N Y N* (2016) 36:689–695. doi: 10.1016/j.nutres.2016.02.012

68. Roh K, Kim S, Kang H, Ku J-M, Park KW, Lee S. Sulfuretin has therapeutic activity against acquired lymphedema by reducing adipogenesis. *Pharmacol Res* (2017) 121:230–239. doi: 10.1016/j.phrs.2017.05.003

69. Roh K, Lee J-H, Kang H, Park KW, Song Y, Lee S, Ku J-M. Synthesis and evaluation of butein derivatives for in vitro and in vivo inflammatory response suppression in lymphedema. *Eur J Med Chem* (2020) 197:112280. doi: 10.1016/j.ejmech.2020.112280

70. Jo M, Trujillo AN, Shibahara N, Breslin JW. The Impact of Goreisan Components on Rat Mesenteric Collecting Lymphatic Vessel Pumping. *Microcirc N Y N 1994* (2023) 30:e12788. doi: 10.1111/micc.12788

71. Cardia GFE, Silva-Filho SE, Silva EL, Uchida NS, Cavalcante HAO, Cassarotti LL, Salvadego VEC, Spironello RA, Bersani-Amado CA, Cuman RKN. Effect of Lavender (Lavandula angustifolia) Essential Oil on Acute Inflammatory Response. *Evid-Based Complement Altern Med ECAM* (2018) 2018:1413940. doi: 10.1155/2018/1413940

72. Essential oils for the lymphatic system. https://www.baseformula.com/blog/aromatherapy-lymphatic-system [Accessed January 19, 2026]

73. Keim AP, Slis JR, Mendez U, Stroup EM, Burmeister Y, Tsolaki N, Gailing O, Goldman J. The Multicomponent Medication Lymphomyosot Improves the Outcome of Experimental Lymphedema. *Lymphat Res Biol* (2013) 11:81–92. doi: 10.1089/lrb.2012.0024

74. Jiang X, Nicolls MR, Tian W, Rockson SG. Lymphatic Dysfunction, Leukotrienes, and Lymphedema. *Annu Rev Physiol* (2018) 80:49–70. doi: 10.1146/annurev-physiol-022516-034008

75. Prangsaengtong O, Jantaree P, Lirdprapamongkol K, Ngiwsara L, Svasti J, Koizumi K. Aspirin suppresses components of lymphangiogenesis and lymphatic vessel remodeling by inhibiting the NF-κB/VCAM-1 pathway in human lymphatic endothelial cells. *Vasc Med Lond Engl* (2018) 23:201–211. doi: 10.1177/1358863X18760718

76. Zhang W, Li J, Liang J, Qi X, Tian J, Liu J. Coagulation in Lymphatic System. *Front Cardiovasc Med* (2021) 8: doi: 10.3389/fcvm.2021.762648

77. Tapia C, Nessel TA, Zito PM. "Cyclosporine.," *StatPearls*. Treasure Island (FL): StatPearls Publishing (2025) http://www.ncbi.nlm.nih.gov/books/NBK482450/ [Accessed January 19, 2026]

78. Ozeki M, Endo S, Yasue S, Nozawa A, Asada R, Saito AM, Hashimoto H, Fujimura T, Yamada Y, Kuroda T, et al. Sirolimus treatment for intractable lymphatic anomalies: an open-label, single-arm, multicenter, prospective trial. *Front Med* (2024) 11: doi: 10.3389/fmed.2024.1335469

79. Gardenier JC, Torrisi JS, Savetsky IL, García Nores GD, Jowhar DK, Hespe GE, Nitti MD, Kataru RP, Mehrara BJ. Topical Tacrolimus for the Treatment of Lymphedema. *J Am Coll Surg* (2015) 221:S120. doi: 10.1016/j.jamcollsurg.2015.07.281
