# Supplementary material for: Pharmacotherapy agents in prevention and treatment of breast cancer-related lymphedema: a systematic scoping review
Source: Front Oncol. 2026 Mar 4;16:1751628. doi: 10.3389/fonc.2026.1751628 (PMC12995638; doi:10.3389/fonc.2026.1751628)
Supplement: Supplementary file 2 [file Table1.docx]

**Supplementary Table 1.** Pharmacological agents, botanicals and dietary supplements and their associated brand name drugs (where available), proposed to influence lymphatic physiology or lymphedema-related pathways, organized by mechanistic similarity. Supplementary table 1A summarizes Venoactive Flavonoids. Supplementary table 1B summarizes herbal and botanical agents grouped by shared mechanistic domains. Supplementary table 1C presents grouped classes of commonly prescribed allopathic anti-inflammatory, anticoagulant, and immunomodulatory medications. Agents without branded counterparts are indicated by “—”. Proposed mechanisms are derived from experimental, translational, or extrapolated clinical literature and do not imply demonstrated efficacy for the prevention or treatment of breast cancer–related lymphedema (BCRL) in cancer survivors.

| **1A.** Venoactive flavonoids. | | | | | | |
| --- | --- | --- | --- | --- | --- | --- |
| **Agent** | | **Brand Name** | **Mechanism of Action Relevant to BCRL** | | | **References in Manuscript** |
| Benzopyrone (Coumarin) | | 56 BAP | Enhances macrophage-mediated proteolysis of interstitial proteins that accumulate due to lymphatic insufficiency. | | | (56) |
| Ruscus aculeatus + Hesperidin methylchalcone | | Cyclo 3 Fort | May enhance lymph propulsion via α-adrenergic–mediated contraction of smooth muscle. | | | (57) |
| Diosmin + Hesperidin (MPFF) | | Daflon | Increases lymphangion contractility and pumping frequency, thereby enhancing lymph flow. | | | (58) |
| Ginko biloba | | BN 165 | May exert antioxidant and anti-inflammatory effects that may protect endothelial and lymphatic vessel integrity. | | | (57) |
| **1B.** Herbal and botanical agents grouped by shared mechanistic domains. | | | | | | |
| **Agent** | **Brand Name** | | | **Mechanism of Action Relevant to BCRL** | **References** | |
| Ruscus aculeatus (Butcher’s broom) | — | | | Proposed to enhance venous–lymphatic tone or lymphatic pumping.   \|  \| \| --- \| | (56,58–63) | |
| Diosmin |  |  |  |  |  |  |
| Hesperidin |  |  |  |  |  |  |
| Diosmetin 7-O-rutinoside |  |  |  |  |  |  |
| Troxerutin |  |  |  |  |  |  |
| Aesculus hippocastanum (Horse chestnut) |  |  |  |  |  |  |
| Cupressus sempervirens (Cypress) |  |  |  |  |  |  |
| Melilotus officinalis (Sweet clover) |  |  |  |  |  |  |
| Vitis vinifera (Grape seed extract) | — | | | Agents targeting capillary permeability, endothelial dysfunction, and microcirculatory inflammation. | (57,64–66) | |
| Olea europaea (Hydroxytyrosol) |  |  |  |  |  |  |
| Quercus robur (Oak) extract |  |  |  |  |  |  |
| Centella asiatica |  |  |  |  |  |  |
| Sodium selenite |  |  |  |  |  |  |
| Scutellaria root |  |  |  |  |  |  |
| Achillea millefolium (Yarrow) |  |  |  |  |  |  |
| Gentiana lutea |  |  |  |  |  |  |
| Paeoniae rubra + Astragalus | — | | | Proposed to modulate inflammation, oxidative stress, or fibrosis. | (57,67–69) | |
| Ginseng |  |  |  |  |  |  |
| Sulfuretin |  |  |  |  |  |  |
| Butein |  |  |  |  |  |  |
| Rhus verniciflua |  |  |  |  |  |  |
| Scrophularia nodosa |  |  |  |  |  |  |
| Colchicine |  |  |  |  |  |  |
| Piper nigrum (Black pepper) |  |  |  |  |  |  |
| Juniper spp. | — | | | Agents with primarily diuretic or fluid-shifting effects. | (57,70) | |
| Alisma rhizome |  |  |  |  |  |  |
| Polyporus sclerotium |  |  |  |  |  |  |
| Poria sclerotium |  |  |  |  |  |  |
| Equisetum hyemale |  |  |  |  |  |  |
| Veronica officinalis |  |  |  |  |  |  |
| Foeniculum vulgare (Fennel) |  |  |  |  |  |  |
| Nasturtium officinale |  |  |  |  |  |  |
| Atractylodes lancea rhizome |  |  |  |  |  |  |
| Lavandula angustifolia (Lavender) | — | | | Agents with topical, aromatic, or nonspecific anti-inflammatory effects | (57,71–73) | |
| Melaleuca alternifolia (Tea tree) |  |  |  |  |  |  |
| Origanum majorana (Marjoram) |  |  |  |  |  |  |
| Pinus sylvestris |  |  |  |  |  |  |
| Geranium spp. |  |  |  |  |  |  |
| Teucrium scorodonia |  |  |  |  |  |  |
| Fumaria officinalis |  |  |  |  |  |  |
| Sarsaparilla |  |  |  |  |  |  |
| Jujube (Ziziphus jujuba) |  |  |  |  |  |  |
| Kampo formulas | — | | | Agents without a meaningful or appropriate lymphedema mechanism | — | |
| Unguentum lymphaticus |  |  |  |  |  |  |
| Glycyrrhiza (Licorice) |  |  |  |  |  |  |
| Hyoscyamine |  |  |  |  |  |  |
| Digitalis |  |  |  |  |  |  |
| Podophyllin |  |  |  |  |  |  |
| Calmodulin |  |  |  |  |  |  |
| Pinellia tuber |  |  |  |  |  |  |
| Angelica sinensis |  |  |  |  |  |  |
| Bupleurum root |  |  |  |  |  |  |
| Tara spinosa |  |  |  |  |  |  |
| **1C.** Grouped classes of commonly prescribed allopathic anti-inflammatory, anti-coagulant, and immunomodulatory medications. | | | | | | |
| **Agent** | **Brand Name** | | | **Mechanism of Action Relevant to BCRL** | **References** | |
| **NSAIDs (****Acetylsalicylic acid, Ketoprofen, Ibuprofen)** | Aspirin, Bayer, Alka-Seltzer, Bufferin, CardioAspirina, Ecotrin, Dispirin, Sedergine, Durlaza, Vazalore, Polopiryna, Orudis, Oruvail, Fastum, Profenid, Bi-Profenid, Flexen, Ketonal, Ketum, Kaltrofen, Diractin, Keplat, Advil, Motrin, Nurofen, Brufen, Caldolor, Moment, Froben, Gelufene, Dolgit, Burana, Algoflex, | | | Inhibit cyclooxygenase (COX-1/COX-2) with effects characterized as primarily symptomatic and anti-inflammatory. | (49,74,75) | |
| **Direct Oral Anti-coagulants (Apixaban, Rivaroxaban)** | Eliquis, Xarelto | | | Anti-inflammatory, anti-coagulant, and immunomodulatory agents may influence lymphatic function indirectly by reducing inflammation, endothelial activation, thrombosis, or fibrotic remodeling. | (76) | |
| **Indirect Factor Xa Inhibitors (Fondaparinux, LMWH class, Dalteparin, Enoxaparin)** | Savaysa, Lixiana, Fragmin, Lovenox, Clexane, Inhixa, Klexane, Ledraxen, Arovi | | |  |  |  |
| **Heparin** | Liquemin, Liquaemin, Hep-Lock, Heparin Leo, Thrombophob, Vetren, Hepalean | | |  |  |  |
| **Parenteral Direct Thrombin Inhibitors (Argatroban, Bivalirudin, Desirudin)** | Novastan, Acova, Exembol, Angiomax, Angiox, Iprivask, Revasc | | |  |  |  |
| **Vitamin K Antagonists (Warfarin)** | Coumadin, Jantoven, Marevan, Waran, Panwarfin | | |  |  |  |
| **Cyclosporine** | Sandimmune, Neoral, Gengraf, Restasis, Cequa, Ikervis, Verkazia, Optimmune | | | Suppresses T-cell activation and inflammatory cytokine production. | (77) | |
| **Hydroxychloroquine** | Quensyl | | | No meaningful or appropriate lymphedema mechanism. | — | |
| **Tacrolimus and Sirolimus** | Prograf, Advagraf, Envarsus XR, Protopic, Modigraf, Rapamune, Fyarro, Hyftor | | | Inhibits lymphatic endothelial cell proliferation, modulating lymphangiogenesis. | (78,79) | |
