# Supplementary material for: Pharmacotherapy agents in prevention and treatment of breast cancer-related lymphedema: a systematic scoping review
Source: Front Oncol. 2026 Mar 4;16:1751628. doi: 10.3389/fonc.2026.1751628 (PMC12995638; doi:10.3389/fonc.2026.1751628)
Supplement: Supplementary file 3 [file Table2.docx]

**Supplementary Table 2.** Search methods and yield for the systematic review: electronic databases searched, platforms, search dates, and records retrieved (including duplicates). Supplemental table 2A summarizes database-level results; Supplemental table sections B–F report the complete, database-specific search strategies for PubMed, Embase, CINAHL, Scopus, and Web of Science, respectively.

| **2A.** Electronic database search summary | | | | |
| --- | --- | --- | --- | --- |
| **Database** | **Platform** | **Search date** | **Full search strategy** | **Records retrieved, n (duplicates)** |
| PubMed | U.S. National Library of Medicine | 05/28/2025 | Supplementary Table 2B. | 96 (0) |
| Embase | Elsevier | 06/02/2025 | Supplementary Table 2C. | 140 (46) |
| Cumulative Index to Nursing and Allied Health Literature | EBSCOhost | 06/02/2025 | Supplementary Table 2D. | 25 (13) |
| Scopus | Elsevier | 06/03/2025 | Supplementary Table 2E. | 34 (16) |
| Web of Science | Clarivate Analytics | 06/05/2025 | Supplementary Table 2F. | 23 (15) |
| **2B.** PubMed search strategy | | | | |
| (56 BaP OR benzopyrone OR coumarin OR cyclo 3 fort OR "cyclo 3 fort" OR hesperidin methylchalcone OR ruscus aculeatus OR butcher's-broom OR daflon OR diosmin OR diosmetin 7-o-rutinoside OR hesperidin OR "BN 165" OR ginkgo biloba OR troxerutin OR foeniculum vulgare OR fennel OR salvia officinalis OR sage OR geranium OR juniper OR black pepper OR piper nigrum OR  oak OR quercus robur OR robuvit OR kampo OR goreisan OR goshajinkigan OR linfadren OR arbutin OR weiyang huoxue washin OR zingiber officinale rosc OR cinnamomum OR zanthoxylum OR angelica sinensis OR sairei to OR chai-ling-tang OR bupleurum root OR pinellia tuber OR alisma rhizome OR scutellaria root OR ginseng OR poria sclerotium OR polyporus sclerotium OR  atractylodes lancea rhizome OR jujube OR glycyrrhiza OR licorice OR cinnamon OR saikosaponin OR lypmhomyosot OR gentiana lutea OR pinus sylvestris OR scrophularia nodosa OR equisetum hyemale OR fumaria officianlis OR geranium robertianum OR nasturtium officinale OR sarsaparilla OR myositis arvensis OR teucrium scorodonia OR veronica officianalis  OR sulfuretin OR rhus verniciflua stokes OR butein OR juzentaihoto OR tara spinosa OR caesalpinia spinosa OR achillea millefolium OR centella asiatica OR olivari OR olive OR olea europaea OR "3,4-dihydroxyphenyl ethanol" OR hydroxytyrosol OR wu ling san OR rhizoma alismatis OR polyporus OR phizoma atractylodes macrocephala OR vitis vinifera OR melaleuca alternifolia OR lavandula angustifolia OR cupressus sempervirens OR origanum majorana OR horse chestnut OR aesculus hippocastanum OR  unguentum lymphaticus OR calmodulin OR hyoscyamine OR digitalis OR cholchicine OR podophyllin OR melilotus officianlis)) OR ("Phytotherapy"[Mesh] OR herb therapy OR herbal therapy) OR ("Herbal Medicine"[Mesh] OR Herbalism OR Hawaiian Herbal Medicine OR "Laau Lapaau" OR "Laau Lapaau" OR "La'au Lapa'au") OR ("Plants, Medicinal"[Mesh] OR Medicinal Plant OR Medicinal Plants OR Medicinal Herbs OR Medicinal Herb OR Pharmaceutical Plants OR Pharmaceutical Plant OR Healing Plants OR Healing Plant) OR ("Dietary Supplements"[Mesh] OR Dietary Supplement OR Dietary Supplementations OR Food Supplementations OR Food Supplements OR Food Supplement OR Nutraceuticals OR Nutraceutical OR Nutriceuticals OR Nutriceutical OR Neutraceuticals OR Neutraceutical OR Herbal Supplements OR Herbal Supplement)) OR  ("Aspirin"[Mesh] OR 2-(Acetyloxy)benzoic Acid OR Acetylsalicylic Acid OR Solprin OR Polopirin OR Solupsan OR Easprin OR Zorprin OR Acetysal OR Acylpyrin OR Colfarit OR Ecotrin OR Endosprin OR Magnecyl OR Micristin OR Polopiryna OR Aloxiprimum OR Dispril) OR  ("Anti-Inflammatory Agents, Non-Steroidal"[Mesh] OR Non-Steroidal Anti-Inflammatory Agent OR Non Steroidal Anti Inflammatory Agent OR NSAID OR NSAIDs OR Nonsteroidal Anti-Inflammatory Agent OR Nonsteroidal Anti Inflammatory Agent OR Nonsteroidal Antiinflammatory Agents OR Non-Steroidal Anti-Inflammatory Agents OR Non Steroidal Anti Inflammatory Agents OR Nonsteroidal Anti-Inflammatory Agents OR Nonsteroidal Anti Inflammatory Agents OR Anti-Inflammatory Analgesics OR Aspirin-Like Agents OR Aspirin Like Agents OR Aspirin-Like Agent OR Aspirin Like Agent) OR  ("Ketoprofen"[Mesh] OR "2-(3-Benzoylphenyl)propionic Acid OR Benzoylhydratropic Acid" OR Alrheumum OR Orudis OR Profenid OR "19,583 RP" OR "RP, 19,583" OR "RP-19583" OR "RP19583" OR "RP 19583" OR Alrheumat) OR ("Ibuprofen"[Mesh] OR ibuprofen OR "alpha-Methyl-4-(2-methylpropyl)benzeneacetic Acid OR Benzeneacetic Acid, alpha-methyl-4-(2-methylpropyl)- trimethylsilyl ester" OR Ibuprofen-Zinc OR Ibuprofen Zinc OR Salprofen OR Brufen OR Motrin OR Ibumetin OR Nuprin OR Rufen OR Trauma-Dolgit Gel OR Trauma Dolgit Gel OR Advil) OR  ("Anticoagulants"[Mesh] OR Anticoagulant Drug OR Anticoagulant Agent OR Anticoagulation Agents OR Anticoagulant Drugs OR Anticoagulant Agents OR Anticoagulant OR Indirect Thrombin Inhibitors) OR ("apixaban"[Supplementary Concept] OR Eliquis OR "BMS 562247" OR "BMS-562247" OR "BMS562247" OR "BMS-562247-01") OR ("Rivaroxaban"[Mesh] OR "BAY 59 7939" OR Xarelto OR "5-chloro-N-(((5S)-2-oxo-3-(4-(3-oxomorpholin-4-yl)phenyl)-1,3-oxazolidin-5-yl)methyl)thiophene-2-carboxamide" OR "BAY 59-7939" OR "BAY 597939") OR  ("edoxaban"[Supplementary Concept] OR Edoxaban OR "N-(5-chloropyridin-2-yl)-N'-((1S,2R,4S)-4-(N,N-dimethylcarbamoyl)-2-(5-methyl-4,5,6,7- tetrahydro(1,3)thiazolo(5,4-c)pyridine-2-carboxamido)cyclohexyl)oxamide" OR "DU-176b" OR "DU-176" OR edoxaban tosylate OR "N-(5-chloropyridin-2-yl)-N'-((1S,2R,4S)-4-(N,N-dimethylcarbamoyl)-2-(5-methyl-4,5,6,7-tetrahydrothiazolo(5,4-c)pyridine-2-carboxamido)cyclohexyl)ethanediamide p-toluenesulfonate monohydrate" OR Savaysa) OR  ("Fondaparinux"[Mesh] OR Fondaparinux OR Fondaparinux Sodium OR Arixtra OR Quixidar) OR ("Heparin"[Mesh] OR Heparin OR Low Molecular Weight Heparin OR Low-Molecular Weight Heparin OR LMWH OR Heparinic Acid OR Unfractionated Heparin OR Sodium Heparin OR Heparin Sodium OR alpha-Heparin OR alpha Heparin OR Liquaemin) OR ("Dalteparin"[Mesh] OR Dalteparin OR Tedelparin OR Dalteparin Sodium OR Fragmin OR Fragmine OR "Kabi-2165" OR "Kabi2165" OR "Kabi 2165" OR "FR-860" OR "FR860" OR "FR 860") OR ("Enoxaparin"[Mesh] OR Enoxaparin OR Enoxaparine OR "EMT-966" OR "EMT966" OR "EMT 966" OR "EMT-967" OR "EMT967" OR "EMT 967" OR Clexane OR "PK-10,169" OR "PK 10,169" OR "PK10,169" OR "PK-10169" OR "PK10169" OR "PK 10169" OR Lovenox) OR ("argatroban" [Supplementary Concept] OR Argatroban OR "2R,4R)-1-(5-((aminoiminomethyl)amino)-1-oxo-2-(((1,2,3,4-tetrahydro-3-methyl-8-quinolinyl)sulfonyl)amino)pentyl)-4-methyl-2-piperidinecarboxylic acid monohydrate OR MMTQAP OR MPQA" OR argatroban monohydrate OR argatroban hydrate OR "MD 805" OR "MD-805" OR "MD805" OR Acova OR Novastan OR argatroban anhydrous OR "(2R,4R)-1-(5-((aminoiminomethyl)amino)-1-oxo-2-(((1,2,3,4-tetrahydro-3-methyl-8-quinolinyl)sulfonyl)amino)pentyl)-4-methyl-2-piperidinecarboxylic acid" OR "(21R)-argatroban anhydrous" OR "(2R,4R)-1-((2S)-5-((aminoiminomethyl)amino)-1-oxo-2-((((3R)-1,2,3,4-tetrahydro-3-methyl-8-quinolinyl)sulfonyl)amino)pentyl)-4-methyl-2-piperidinecarboxylic acid" OR "(21S)-argatroban anhydrous PR (2R,4R)-1-((2S)-5-((aminoiminomethyl)amino)-1-oxo-2-((((3S)-1,2,3,4-tetrahydro-3-methyl-8-quinolinyl)sulfonyl)amino)pentyl)-4-methyl-2-piperidinecarboxylic acid" OR "(21S)-argatroban" OR "MCI 9038" OR "MCI-9038" OR (21R)-argatroban) OR ("bivalirudin" [Supplementary Concept] OR bivalirudin OR "L-leucine, D-phenylalanyl-L-prolyl-L-arginyl-L-prolylglycylglycylglycylglycyl-L-asparaginylglycyl-L-alpha-aspartyl-L-phenylalanyl-L-alpha-glutamyl-L-alpha-glutamyl-L-isoleucyl-L-prolyl-L-alpha-glutamyl-L-alpha-glutamyl-L-tyrosyl- OR Phe-Pro-Arg-Pro-(Gly)4 desulfato-Tyr63'-hirugen" OR "Phe-Pro-Arg-Pro-(Gly)4-Asn-Gly-Asp-Phe-Glu-Glu-Ile-Pro-Glu-Glu-Tyr-Leu OR Phe-Pro-Arg-Pro-(Gly)4-desulfohirudin-(53-64)" OR "BG 8967" OR "BG-8967" OR "BG8967" OR Hirulog OR Hirulog-1 OR Angiomax OR Angiomax RTU OR "CTB-001" OR bivalirudin trifluoroacetate) OR ("Dabigatran"[Mesh] OR Dabigatran OR "N-((2-(((4-(aminoiminomethyl)phenyl)amino)methyl)-1-methyl-1H-benzimidazol-5-yl)carbonyl)-N-2-pyridinyl-beta-alanine" OR Dabigatran Etexilate OR "BIBR 1048" OR Pradaxa OR Dabigatran Etexilate Mesylate) OR ("desirudin" [Supplementary Concept] OR Desirudin OR desulfatohirudin OR desulphatohirudin OR recombinant HV1 hirudin OR "CGP 39393" OR "CGP-39393" OR "IK-HIR02" OR Revasc OR Iprivask) OR ("Warfarin"[Mesh] OR Warfarin OR Coumadin OR "4-Hydroxy-3-(3-oxo-1-phenylbutyl)-2H-1-benzopyran-2-one" OR Warfarin Potassium OR Warfarin Sodium OR Marevan OR Gen-Warfarin OR Aldocumar OR Coumadine OR Apo-Warfarin OR Tedicumar OR Warfant) OR ("Cyclosporine"[Mesh] OR Cyclosporin OR Ciclosporin OR Cyclosporin OR Cyclosporin A OR Cyclosporine A OR CsA-Neoral OR CsANeoral OR CsA Neoral OR Sandimmun Neoral OR CyA-NOF OR CyA NOF OR Sandimmune OR Sandimmun OR Neoral OR "OL 27-400" OR "OL 27400" OR "OL 27 400") OR ("Hydroxychloroquine"[Mesh] OR hydroxychloroquine OR Hydroxychlorochin OR Oxychloroquine OR Oxychlorochin OR Plaquenil OR Hydroxychloroquine Sulfate OR Hydroxychloroquine Sulfate (1:1) Salt) OR ("Tacrolimus"[Mesh] OR Tacrolimus OR Prograft OR Anhydrous Tacrolimus OR "FK-506" OR "FK 506" OR "FK506" OR "FR-900506" OR "FR900506" OR "FR 900506") OR ("Sirolimus"[Mesh] OR Sirolimus OR Rapamycin OR Rapamune OR "AY 22-989" OR "AY 22989" OR "AY 22 989" OR "I-2190A" OR "I2190A" OR "I 2190A") OR (Leukotriene B4 antagonist OR Leukotriene B4 inhibitor) OR ("Leukotriene B4"[Mesh] OR Leukotriene B4 OR Leukotriene B-4 OR "Leukotriene B 4" OR LTB4 OR "5,12-diHETE" OR "5,12 diHETE" OR "5,12-HETE" OR "5,12 HETE" OR "Leukotrienes B" OR "Leukotriene B") OR ("Platelet-Rich Plasma"[Mesh] OR Platelet Rich Plasma OR Platelet-Rich Plasma) OR ("Immunosuppressive Agents"[Mesh] OR Immunosuppressive Agent OR Immunosuppressant OR Immunosuppressants) AND ("Breast Cancer Lymphedema"[Mesh] OR Breast Cancer Lymphedemas OR Breast Cancer Related Lymphedema OR Breast Cancer Treatment-Related Lymphedema OR Breast Cancer Treatment Related Lymphedema OR Breast Cancer-Related Arm Lymphedema OR Breast Cancer Related Arm Lymphedema OR Postmastectomy Lymphedema OR Postmastectomy Lymphedemas OR Post-mastectomy Lymphedema OR Post mastectomy Lymphedema OR Post-mastectomy Lymphedemas)) | | | | |
| **2C.** Embase search strategy | | | | |
| ('56 bap' OR (56 AND bap) OR 'benzopyrone'/exp OR benzopyrone OR 'coumarin'/exp OR coumarin OR (cyclo AND ('3'/exp OR 3) AND fort) OR 'cyclo 3 fort'/exp OR 'cyclo 3 fort' OR 'hesperidin methylchalcone'/exp OR 'hesperidin methylchalcone' OR (('hesperidin'/exp OR hesperidin) AND methylchalcone) OR 'ruscus aculeatus'/exp OR 'ruscus aculeatus' OR (('ruscus'/exp OR ruscus) AND aculeatus) OR 'butchers broom' OR 'daflon'/exp OR daflon OR 'diosmin'/exp OR diosmin OR 'diosmetin 7-o-rutinoside' OR (('diosmetin'/exp OR diosmetin) AND '7 o rutinoside') OR 'hesperidin'/exp OR hesperidin OR 'bn 165' OR 'ginkgo biloba'/exp OR 'ginkgo biloba' OR (('ginkgo'/exp OR ginkgo) AND biloba) OR 'troxerutin'/exp OR troxerutin OR 'foeniculum vulgare'/exp OR 'foeniculum vulgare' OR (('foeniculum'/exp OR foeniculum) AND vulgare) OR 'fennel'/exp OR fennel OR 'salvia officinalis'/exp OR 'salvia officinalis' OR (('salvia'/exp OR salvia) AND officinalis) OR 'sage'/exp OR sage OR 'geranium'/exp OR geranium OR 'juniper'/exp OR juniper OR 'black pepper'/exp OR 'black pepper' OR (('black'/exp OR black) AND ('pepper'/exp OR pepper)) OR 'piper nigrum'/exp OR 'piper nigrum' OR (('piper'/exp OR piper) AND nigrum) OR 'oak'/exp OR oak OR 'quercus robur'/exp OR 'quercus robur' OR (('quercus'/exp OR quercus) AND robur) OR 'robuvit'/exp OR robuvit OR 'kampo'/exp OR kampo OR 'goreisan'/exp OR goreisan OR 'goshajinkigan'/exp OR goshajinkigan OR linfadren OR 'arbutin'/exp OR arbutin OR 'weiyang huoxue washin' OR (weiyang AND ('huoxue'/exp OR huoxue) AND washin) OR 'zingiber officinale rosc' OR (('zingiber'/exp OR zingiber) AND officinale AND ('rosc'/exp OR rosc)) OR 'cinnamomum'/exp OR cinnamomum OR 'zanthoxylum'/exp OR zanthoxylum OR 'angelica sinensis'/exp OR 'angelica sinensis' OR (('angelica'/exp OR angelica) AND sinensis) OR 'sairei to'/exp OR 'sairei to' OR (sairei AND to) OR 'chai ling tang' OR 'bupleurum root'/exp OR 'bupleurum root' OR (('bupleurum'/exp OR bupleurum) AND ('root'/exp OR root)) OR 'pinellia tuber'/exp OR 'pinellia tuber' OR (('pinellia'/exp OR pinellia) AND ('tuber'/exp OR tuber)) OR 'alisma rhizome' OR (('alisma'/exp OR alisma) AND ('rhizome'/exp OR rhizome)) OR 'scutellaria root' OR (('scutellaria'/exp OR scutellaria) AND ('root'/exp OR root)) OR 'ginseng'/exp OR ginseng OR 'poria sclerotium'/exp OR 'poria sclerotium' OR (('poria'/exp OR poria) AND ('sclerotium'/exp OR sclerotium)) OR 'polyporus sclerotium' OR (('polyporus'/exp OR polyporus) AND ('sclerotium'/exp OR sclerotium)) OR 'atractylodes lancea rhizome'/exp OR 'atractylodes lancea rhizome' OR (('atractylodes'/exp OR atractylodes) AND lancea AND ('rhizome'/exp OR rhizome)) OR 'jujube'/exp OR jujube OR 'glycyrrhiza'/exp OR glycyrrhiza OR 'licorice'/exp OR licorice OR 'cinnamon'/exp OR cinnamon OR 'saikosaponin'/exp OR saikosaponin OR lypmhomyosot OR 'gentiana lutea'/exp OR 'gentiana lutea' OR (('gentiana'/exp OR gentiana) AND lutea) OR 'pinus sylvestris'/exp OR 'pinus sylvestris' OR (('pinus'/exp OR pinus) AND sylvestris) OR 'scrophularia nodosa'/exp OR 'scrophularia nodosa' OR (('scrophularia'/exp OR scrophularia) AND nodosa) OR 'equisetum hyemale'/exp OR 'equisetum hyemale' OR (('equisetum'/exp OR equisetum) AND hyemale) OR 'fumaria officianlis' OR (('fumaria'/exp OR fumaria) AND officianlis) OR 'geranium robertianum'/exp OR 'geranium robertianum' OR (('geranium'/exp OR geranium) AND robertianum) OR 'nasturtium officinale'/exp OR 'nasturtium officinale' OR (('nasturtium'/exp OR nasturtium) AND officinale) OR 'sarsaparilla'/exp OR sarsaparilla OR 'myositis arvensis' OR (('myositis'/exp OR myositis) AND arvensis) OR 'teucrium scorodonia' OR (('teucrium'/exp OR teucrium) AND scorodonia) OR 'veronica officianalis' OR (('veronica'/exp OR veronica) AND officianalis) OR 'sulfuretin'/exp OR sulfuretin OR 'rhus verniciflua stokes' OR (('rhus'/exp OR rhus) AND verniciflua AND stokes) OR 'butein'/exp OR butein OR 'juzentaihoto'/exp OR juzentaihoto OR 'tara spinosa'/exp OR 'tara spinosa' OR (tara AND spinosa) OR 'caesalpinia spinosa'/exp OR 'caesalpinia spinosa' OR (('caesalpinia'/exp OR caesalpinia) AND spinosa) OR 'achillea millefolium'/exp OR 'achillea millefolium' OR (('achillea'/exp OR achillea) AND millefolium) OR 'centella asiatica'/exp OR 'centella asiatica' OR (('centella'/exp OR centella) AND asiatica) OR olivari OR 'olive'/exp OR olive OR 'olea europaea'/exp OR 'olea europaea' OR (('olea'/exp OR olea) AND europaea) OR '3,4-dihydroxyphenyl ethanol'/exp OR '3,4-dihydroxyphenyl ethanol' OR 'hydroxytyrosol'/exp OR hydroxytyrosol OR 'wu ling san'/exp OR 'wu ling san' OR (wu AND ling AND san) OR 'rhizoma alismatis'/exp OR 'rhizoma alismatis' OR (('rhizoma'/exp OR rhizoma) AND ('alismatis'/exp OR alismatis)) OR 'polyporus'/exp OR polyporus OR 'phizoma atractylodes macrocephala' OR (phizoma AND ('atractylodes'/exp OR atractylodes) AND macrocephala) OR 'vitis vinifera'/exp OR 'vitis vinifera' OR (('vitis'/exp OR vitis) AND vinifera) OR 'melaleuca alternifolia'/exp OR 'melaleuca alternifolia' OR (('melaleuca'/exp OR melaleuca) AND alternifolia) OR 'lavandula angustifolia'/exp OR 'lavandula angustifolia' OR (('lavandula'/exp OR lavandula) AND angustifolia) OR 'cupressus sempervirens'/exp OR 'cupressus sempervirens' OR (('cupressus'/exp OR cupressus) AND sempervirens) OR 'origanum majorana'/exp OR 'origanum majorana' OR (('origanum'/exp OR origanum) AND majorana) OR 'horse chestnut'/exp OR 'horse chestnut' OR (('horse'/exp OR horse) AND ('chestnut'/exp OR chestnut)) OR 'aesculus hippocastanum'/exp OR 'aesculus hippocastanum' OR (('aesculus'/exp OR aesculus) AND hippocastanum) OR 'unguentum lymphaticus' OR (('unguentum'/exp OR unguentum) AND lymphaticus) OR 'calmodulin'/exp OR calmodulin OR 'hyoscyamine'/exp OR hyoscyamine OR 'digitalis'/exp OR digitalis OR cholchicine OR 'podophyllin'/exp OR podophyllin OR 'melilotus officianlis' OR (('melilotus'/exp OR melilotus) AND officianlis) OR ((('phytotherapy'/exp OR herb) AND therapy OR herbal) AND therapy) OR ((('herbal medicine'/exp OR herbalism OR hawaiian) AND herbal AND medicine OR laau) AND lapaau) OR 'medicinal plant'/exp OR 'medicinal herb' OR 'medicinal herbs' OR 'pharmaceutical plant' OR 'pharmaceutical plants' OR 'healing plant' OR 'healing plants' OR 'dietary supplement'/exp OR 'diet supplementation' OR 'diet supplement' OR 'nutraceutical'/exp OR 'nutraceutical' OR 'nutriceutical' OR 'nutriceuticals' OR 'food supplementation' OR 'food supplement' OR 'food supplementations' OR 'dietary supplementations' OR 'herbal supplement' OR 'herbal supplementations' OR 'herbal supplementation' OR 'ethnobotanical medicine' OR 'phyto-medical remedy' OR 'botanical medicine' OR  'acetylsalicylic acid'/exp OR '2 acetoxybenzoate' OR '2 acetoxybenzoic acid' OR '8-hour bayer' OR 'acenterine' OR 'acesal' OR 'acetan' OR 'acetard' OR 'aceticil' OR 'aceticyl' OR 'acetilum' OR 'acetonyl' OR 'acetophen' OR 'acetosal' OR 'acetosalicylic acid' OR 'acetosalin' OR 'acetosalum' OR 'acetyl salicylate' OR 'acetyl salicylic acid' OR 'acetylic salicylic acid' OR 'acetylin' OR 'acetylo' OR 'acetylo salicylic acid' OR 'acetylon' OR 'acetylosalicylic acid' OR 'acetylsal' OR 'acetylsalicyclic acid' OR 'acetylsalicyl' OR 'acetylsalicylate' OR 'acetylsalicylate strontium' OR 'acetylsalicylic acid plus glycine' OR 'acetylsalicylic acid sodium salt' OR 'acetylsalicylic acid strontium salt' OR 'acetylsalycic acid' OR 'acetylsalycylic acid' OR 'acetysal' OR 'acidulatum' OR 'acidum acetyl salicylicum' OR 'acidum acetylosalicylicum' OR 'acidum acetylsalicylicum' OR 'actorin' OR 'acylpyrin' OR 'acylpyrine' OR 'acytosal' OR 'adiro' OR 'alabukun' OR 'alasil' OR 'albyl e' OR 'albyl minor' OR 'albyl-e' OR 'alka seltzer' OR 'alka-seltzer' OR 'alkaspirin' OR 'anasprin' OR 'andol' OR 'anopyrin' OR 'ansin' OR 'anthrom' OR 'aptor' OR 'arthralgyl' OR 'arthritis strength bufferin' OR 'asa' OR 'asa akut' OR 'asa cardio' OR 'asa direk' OR 'asa effect' OR 'asa express' OR 'asa migraene' OR 'asa migrane' OR 'asa migren' OR 'asa pro' OR 'asa protect' OR 'asa ultra' OR 'asa ultra fast' OR 'asa zipp' OR 'asaa' OR 'asaa gr' OR 'asaa microactive' OR 'asaa rapida' OR 'asacard' OR 'asae' OR 'asae bruis' OR 'asae ec protect' OR 'asae fasttabs' OR 'asae protect' OR 'asaetta' OR 'asaflow' OR 'asaphen' OR 'asaphen e.c.' OR 'asapor' OR 'asatard' OR 'asawin' OR 'aspec' OR 'aspec-ec' OR 'aspent' OR 'aspergum' OR 'aspex' OR 'aspilets' OR 'aspirem' OR 'aspirgran' OR 'aspiricor' OR 'aspirin' OR 'aspirin bayer' OR 'aspirina' OR 'aspirine' OR 'aspirinine' OR 'aspirisucre' OR 'aspisol' OR 'aspo cid' OR 'aspro' OR 'aspro cardio' OR 'aspro clear' OR 'asproflash' OR 'asrina' OR 'asrivo' OR 'asta' OR 'asteric' OR 'asteric acid' OR 'astrix' OR 'bamyl' OR 'bayaspirina' OR 'bayer aspirin' OR 'bayer aspirin cardio' OR 'bayer extra strength aspirin for migraine pain' OR 'bebesan' OR 'biprin' OR 'bokey' OR 'boxazin' OR 'breoprin' OR 'buffered aspirin' OR 'bufferin' OR 'bufferin low dose' OR 'cafenol' OR 'caprin (acetylsalicylic acid)' OR 'caprin (aspirin)' OR 'cardioasa' OR 'cardioasae' OR 'cardioaspirina' OR 'cardioflow (acetylsalicylic acid)' OR 'cartia' OR 'caspirin' OR 'catalgine' OR 'catalgix' OR 'cemerit' OR 'cemirit' OR 'claradin' OR 'claragine' OR 'colfarit' OR 'comoprin' OR 'contrheuma' OR 'contrheuma retard' OR 'darosal' OR 'depot aspirin' OR 'dispirin' OR 'dolean' OR 'durlaza' OR 'dusil' OR 'easprin' OR 'ecasil' OR 'ecosprin' OR 'ecotrin' OR 'ecotrin 650' OR 'egalgic' OR 'emocin' OR 'empirin' OR 'encaprin' OR 'encine em' OR 'endosprin' OR 'entaprin' OR 'entericin' OR 'enteroprin' OR 'enterosarine' OR 'enterospirine' OR 'entrophen' OR 'eskotrin' OR 'euthermine' OR 'extren' OR 'flamasacard' OR 'genasprin' OR 'globentyl' OR 'godamed' OR 'gotosan' OR 'helicon' OR 'herz ass' OR 'hjertemagnyl' OR 'idotyl' OR 'infatabs a' OR 'istopirin' OR 'istopyrine' OR 'ivepirine' OR 'juvepirine' OR 'keypo' OR 'kilios' OR 'kinderaspirin' OR 'magnecyl brus' OR 'magnyl dak' OR 'mcn r 358' OR 'measurin' OR 'mejoral' OR 'melabon' OR 'micristin' OR 'micropyrin' OR 'migrasaa' OR 'mikristin' OR 'miniasal' OR 'mycristin' OR 'naspro' OR 'novasen' OR 'nu seal' OR 'nu-seals' OR 'nu-seals asa' OR 'nuseals' OR 'ortho acetoxybenzoate' OR 'ortho acetoxybenzoic acid' OR 'ortho acetyloxybenzoate' OR 'ortho acetyloxybenzoic acid' OR 'ostoprin' OR 'pancemol' OR 'para acetylsalicylic acid' OR 'paracin' OR 'paynocil' OR 'pengo' OR 'platet 300 cleartab' OR 'plewin' OR 'polopiryna' OR 'premaspin' OR 'primaspan' OR 'proprin' OR 'pyronoval' OR 'reumyl' OR 'rhodine' OR 'rhonal' OR 'rhonal for children' OR 'ronal' OR 'salacetin' OR 'salacetogen' OR 'saletin' OR 'salisalido' OR 'salospir' OR 'sargepirine' OR 'sedergine' OR 'sedergine forte' OR 'slow release aspirin' OR 'sodium acetylsalicylate' OR 'sodium bicarbonate acetyl salicylate' OR 'sodium bicarbonate acetylsalicylate' OR 'soldral' OR 'solpyron' OR 'solucetyl' OR 'solupsa' OR 'spren' OR 'super tru' OR 'tapal' OR 'temagin' OR 'tevapirin' OR 'th 2152' OR 'thrombo-aspilets' OR 'toldex retard' OR 'treupahlin' OR 'treuphalin' OR 'tromalyt' OR 'tromcor' OR 'turivital' OR 'vazalore' OR 'verin' OR 'vitalink' OR 'xaxa' OR 'ysp aspirin' OR 'zero-order release' OR 'zorprin' OR 'acetylsalicylic acid' OR 'nonsteroid antiinflammatory agent'/exp OR 'non-steroidal anti-inflammatory agent' OR 'nsaid' OR 'nsaids' OR 'nonsteroidal anti-inflammatory agent' OR 'nonsteroidal anti inflammatory agent' OR 'nonsteroidal antiinflammatory agents' OR 'non-steroidal anti-inflammatory agents' OR 'non steroidal anti inflammatory agents' OR 'nonsteroidal anti inflammatory agents nonsteroidal' OR 'anti inflammatory agents' OR 'anti inflammatory analgesics' OR 'aspirin like agent' OR 'aspirin like agents' OR 'ketoprofen'/exp OR '19583 rp' OR '2 (3 benzoylphenyl) propionic acid' OR '2 [3 benzoyl (phenyl)] propionic acid' OR 'actron (ketoprofen)' OR 'alreumat' OR 'alrhemun' OR 'alrheumat' OR 'alrheumin' OR 'alrheumun' OR 'alrhumat' OR 'aneol' OR 'anzema' OR 'apo-keto' OR 'arcental' OR 'begsan' OR 'bi-profenid' OR 'bi-profenid lp' OR 'bi-rofenid' OR 'biprofenid' OR 'birofenid' OR 'capisten' OR 'cetoprofen' OR 'diractin' OR 'dolofar' OR 'drastirel' OR 'efiken' OR 'els m11' OR 'elsm11' OR 'epatec' OR 'extraplus (drug)' OR 'fastum' OR 'fetik' OR 'flexen (ketoprofen)' OR 'floramil' OR 'gabrilen' OR 'gabrilen retard' OR 'gesicpad k' OR 'helenil' OR 'ibifen' OR 'idea 033' OR 'idea033' OR 'impracor' OR 'ipi 110' OR 'ipi110' OR 'iso k (drug)' OR 'jomezal' OR 'kaltrofen' OR 'kebanon' OR 'keduril' OR 'kefen' OR 'kehancer' OR 'kenhancer' OR 'kenofen gel' OR 'keotsan' OR 'keplat' OR 'keprofen' OR 'ketadom' OR 'ketin' OR 'keto film' OR 'ketofarm' OR 'ketofen' OR 'ketoflam' OR 'ketolgin' OR 'ketolgin gel' OR 'ketolgin sr' OR 'ketomex' OR 'ketonal' OR 'ketoprofen sodium' OR 'ketoprofene' OR 'ketores' OR 'ketorin' OR 'ketosolan' OR 'ketospray' OR 'ketotransdel' OR 'ketum' OR 'knavon' OR 'kpl 202' OR 'liotondol' OR 'lisactiv gola' OR 'mohrus' OR 'naxal' OR 'nexcede' OR 'novo-keto-ec' OR 'opokan keto' OR 'oralcare (drug)' OR 'orochet' OR 'orucote' OR 'orudis' OR 'orudis e-100' OR 'orudis ec' OR 'orudis kt' OR 'orudis r' OR 'orudis r-pr' OR 'orudis retard' OR 'orudis sr' OR 'oruvail' OR 'oruvail ec' OR 'oruvail sr' OR 'oscorel' OR 'ovurila' OR 'ovurila e' OR 'oxoprofene' OR 'phardol' OR 'profecom' OR 'profemigr' OR 'profenid' OR 'profenid 50' OR 'profenid lp' OR 'profika' OR 'prontoflex' OR 'protofen' OR 'provail cr' OR 'rematof' OR 'rhetoflam' OR 'rheuna pap' OR 'rofenid' OR 'rofixdol' OR 'rp 19, 583' OR 'rp 19583' OR 'siduro' OR 'sodium ketoprofen' OR 'spondylon' OR 'tdlp 110' OR 'tdlp110' OR 'tiloket' OR 'topofen' OR 'toprec' OR 'treosin' OR 'valket' OR 'yerasel' OR 'ketoprofen' OR 'ibuprofen'/exp OR '(4 isobutylphenyl) alpha methylacetic acid' OR '2 (4 isobutylphenyl) propanoic acid' OR '2 (4 isobutylphenyl) propionic acid' OR '2 (para isobutylphenyl) propanoic acid' OR '2 (para isobutylphenyl) propionic acid' OR '2 [4 (2 methylpropyl) phenyl] propanoic acid' OR '2 [4 (2 methylpropyl) phenyl] propionic acid' OR '4 (2 methylpropyl) alpha methylphenylacetic acid' OR '4 (2 methylpropyl) hydratropic acid' OR '4 isobutyl alpha methylphenylacetic acid' OR '4 isobutylhydratropic acid' OR '[4 (2 methylpropanyl) phenyl] alpha methylacetic acid' OR '[4 (2 methylpropyl) phenyl] alpha methylacetic acid' OR 'abfen' OR 'abidol (ibuprofen)' OR 'aches-n-pain' OR 'act-3' OR 'actiprofen' OR 'adagin' OR 'adagin forte' OR 'adex 200' OR 'adex liqui-gels' OR 'adolorin ibuforte' OR 'adolorin ibuforte direkt' OR 'adolorini direkt' OR 'advil' OR 'advil infantil' OR 'advil liqui-gels' OR 'advil liquid caps' OR 'advil liquifast' OR 'advil liquigel' OR 'advil migraine liqui-gels' OR 'advil mono' OR 'advil paediatric' OR 'advil pediatric' OR 'advil reliva forte' OR 'advilcaps' OR 'advileff' OR 'advilgel' OR 'advilmed' OR 'advilmed enfants et nourrissons' OR 'adviltab' OR 'afebril' OR 'aktren' OR 'aktren forte' OR 'aktren mobil' OR 'aktren spezial' OR 'algiasdin' OR 'algiasdin retard' OR 'algifor' OR 'algik (ibuprofen)' OR 'algin baby' OR 'algofen (ibuprofen)' OR 'algoflex' OR 'algoflex dolo' OR 'algoflex forte' OR 'algoflex norma' OR 'algoflex rapid' OR 'algoflex ultra forte' OR 'algofren' OR 'algopirina febbre e dolore' OR 'algopirindol' OR 'algovil' OR 'allipen' OR 'alpha (4 isobutylphenyl) propanoic acid' OR 'alpha (4 isobutylphenyl) propionic acid' OR 'alpha [4 (2 methylpropanyl) phenyl] propanoic acid' OR 'alpha [4 (2 methylpropanyl) phenyl] propionic acid' OR 'alpha [4 (2 methylpropyl) phenyl] propanoic acid' OR 'alpha [4 (2 methylpropyl) phenyl] propionic acid' OR 'alpha methyl 4 (2 methylpropanyl) benzeneacetic acid' OR 'alpha methyl 4 (2 methylpropyl) benzeneacetic acid' OR 'alpha methyl 4 (isobutyl) phenylacetic acid' OR 'alpha methyl 4 isobutylbenzeneacetic acid' OR 'alvofen express' OR 'am-fam 400' OR 'ambaprofen' OR 'anadin (ibuprofen)' OR 'anadin joint pain' OR 'anadin liquifast' OR 'anadin period pain' OR 'anadin ultra' OR 'anadvil' OR 'analgyl' OR 'anbifen' OR 'anco' OR 'andran' OR 'anflagen' OR 'antalfebal' OR 'antalgil' OR 'antarene' OR 'antiflam' OR 'apirofeno' OR 'apo-ibuprofen' OR 'apo-rapid' OR 'aragel' OR 'articalm' OR 'asda long lasting pain relief' OR 'atril 300' OR 'attritin' OR 'auxifen' OR 'axoprofen' OR 'axoprofen forte' OR 'azodermol' OR 'babyfen' OR 'balkaprofen' OR 'bells healthcare children`s pain and fever relief' OR 'bells healthcare period pain relief' OR 'benatia' OR 'berlistar' OR 'berlistar forte' OR 'bestafen' OR 'betaprofen' OR 'bifen' OR 'blokmax' OR 'blokmax forte' OR 'bluton' OR 'brinflan' OR 'brufanic' OR 'brufedol' OR 'brufen' OR 'brufen 400' OR 'brufen bruis' OR 'brufen cukormentes' OR 'brufen effect' OR 'brufen effect forte' OR 'brufen forte' OR 'brufen granules' OR 'brufen instant' OR 'brufen paediatric' OR 'brufen retard' OR 'brufen sr' OR 'brufenact' OR 'brufenlik' OR 'brufort' OR 'brugesic' OR 'brumare (ibuprofen)' OR 'brumed' OR 'brupro' OR 'brupro max' OR 'brupro rx' OR 'brusimp' OR 'buburone' OR 'bufect' OR 'bufect forte' OR 'bufohexal' OR 'buplex' OR 'buplex rx' OR 'bupogesic' OR 'buprofess' OR 'buprophar' OR 'burana' OR 'burana-caps' OR 'buranagel' OR 'buripin' OR 'buscofem' OR 'buscofen' OR 'buscofenact' OR 'buscofenpocket' OR 'butacortelone' OR 'butifen' OR 'caldolor' OR 'calmolin' OR 'calprofen' OR 'cap-profen' OR 'cenbufen' OR 'children`s advil' OR 'children`s advil-flavored' OR 'children`s elixsure' OR 'children`s ibuprofen' OR 'children`s motrin' OR 'childrens motrin' OR 'cibalgina due fast' OR 'cibalginafor' OR 'cinfadol' OR 'codral period pain' OR 'combiflam' OR 'contraneural' OR 'cuprofen' OR 'dalsy' OR 'dalsy forte' OR 'dalsydol' OR 'danofen' OR 'dc 7034' OR 'dc7034' OR 'deliprom' OR 'demaflan' OR 'dg 7034' OR 'dg7034' OR 'dibufen' OR 'dicloreum unidie' OR 'diffutab sr 600' OR 'dimidon' OR 'dismenol' OR 'dismenol forte' OR 'diverin' OR 'doc schmerzgel' OR 'dolan fp' OR 'dolgit' OR 'dolibloc' OR 'dolirief' OR 'dolobene ibu' OR 'dolocyl' OR 'dolodolgit' OR 'dolofen-f' OR 'dolofort' OR 'dolomax' OR 'dolorgiet' OR 'dolowill' OR 'dolval' OR 'donjust b' OR 'dorival' OR 'druisel' OR 'easifon' OR 'easofen' OR 'easofen max strength' OR 'ebufac' OR 'ecoprofen' OR 'eiren' OR 'emflam' OR 'emflam-200' OR 'epobron' OR 'ergix douleur et fievre' OR 'essential waitrose long lasting pain relief' OR 'eudorlin (ibuprofen)' OR 'eudorlin extra' OR 'eudorlin infantil' OR 'eudorlin migrane' OR 'exidol' OR 'expanfen' OR 'extrapan' OR 'febratic' OR 'febrirol' OR 'febryn' OR 'fedotask' OR 'femapirin' OR 'fenalgic' OR 'fenbid' OR 'fenbid forte' OR 'fenopine' OR 'fenpaed' OR 'fevralt' OR 'fiedosin' OR 'flamicon' OR 'flarin' OR 'fluibron febbre e dolore' OR 'fluifort febbre e dolore' OR 'focus (drug)' OR 'forsium' OR 'frenidor' OR 'froben dolore e febbre' OR 'froben dolore e infiammazione' OR 'frobenkids febbre e dolore' OR 'galpharm migraine relief' OR 'galprofen' OR 'galprofen max' OR 'gelib' OR 'gelufene' OR 'gyno-neuralgin' OR 'h-loniten' OR 'halprin' OR 'haltran' OR 'hemagene tailleur' OR 'holuren' OR 'ib-100' OR 'ib-u-ron' OR 'ibacut' OR 'ibalgin' OR 'ibalgin baby' OR 'ibalgin express' OR 'ibalgin forte' OR 'ibalgin gel' OR 'ibalgin instant' OR 'ibalgin junior' OR 'ibalgin krem' OR 'ibalgin neo' OR 'ibalgin rapidcaps' OR 'ibenal' OR 'ibenal forte' OR 'ibenal max' OR 'ibetin' OR 'iblasin' OR 'ibofen' OR 'ibosure' OR 'ibu' OR 'ibu akut' OR 'ibu slow' OR 'ibu-4' OR 'ibu-6' OR 'ibu-8' OR 'ibu-dent' OR 'ibu-puren' OR 'ibu-slow' OR 'ibu-tab' OR 'ibu-tab 200' OR 'ibuaristo akut' OR 'ibubel' OR 'ibuberl' OR 'ibubeta' OR 'ibucalm' OR 'ibucalmin' OR 'ibucaps' OR 'ibudak' OR 'ibuderm' OR 'ibudex' OR 'ibudol' OR 'ibudol pediatrico' OR 'ibudol roll on' OR 'ibudol roll-on' OR 'ibudolor' OR 'ibufarmalid' OR 'ibufem' OR 'ibufen' OR 'ibuflam' OR 'ibufug' OR 'ibugard' OR 'ibugel' OR 'ibugel forte' OR 'ibugesic' OR 'ibuhexal akut' OR 'ibukern' OR 'ibukey' OR 'ibuklaph' OR 'ibuleve' OR 'ibuleve max strength pain relief' OR 'ibuleve maximum strength' OR 'ibuleve pain relief' OR 'ibuleve sports gel' OR 'ibulgan' OR 'ibuloid' OR 'ibum' OR 'ibum femina' OR 'ibum forte' OR 'ibum supermax' OR 'ibumal' OR 'ibumax' OR 'ibumax forte' OR 'ibumed' OR 'ibumenol' OR 'ibumetin' OR 'ibumetin forte' OR 'ibumousse' OR 'ibunid' OR 'ibunin' OR 'ibupar' OR 'ibupar forte' OR 'ibupen' OR 'ibupirac' OR 'ibupradoll' OR 'ibuprex' OR 'ibuprex max' OR 'ibuprin' OR 'ibuprocin' OR 'ibuprofen klinge 600' OR 'ibuprofen potassium' OR 'ibuprofen sodium' OR 'ibuprofene' OR 'ibuprohm' OR 'ibuprom' OR 'ibuprom effect' OR 'ibuprom forte' OR 'ibuprom max' OR 'ibuprom max rapid' OR 'ibuprom max sprint' OR 'ibuprom regular' OR 'ibuprom sport' OR 'ibuprom sport spray' OR 'ibuprom ultramax' OR 'ibuprom ultramax sprint' OR 'iburapid' OR 'ibured' OR 'ibured forte' OR 'iburon' OR 'ibusal' OR 'ibusan' OR 'ibusolve' OR 'ibuspray' OR 'ibustar' OR 'ibustar forte' OR 'ibustick' OR 'ibusynth' OR 'ibutabs' OR 'ibutact' OR 'ibutad' OR 'ibuteva' OR 'ibutop' OR 'ibuvalen' OR 'ibuviva' OR 'ibux' OR 'ibuxin' OR 'idyl sr' OR 'ifenin' OR 'imofen' OR 'infant`s motrin' OR 'infibu' OR 'inflanor' OR 'inflanor forte' OR 'inoflon' OR 'ipren' OR 'iprensa' OR 'irfen' OR 'junifen' OR 'junior strength advil' OR 'junior strength ibuprofen' OR 'junior strength motrin' OR 'junipro' OR 'kendo' OR 'kenfen' OR 'kidofen' OR 'kidofen max' OR 'kontraneural' OR 'lamidon' OR 'larofen' OR 'levifen' OR 'librofem' OR 'liderfeme' OR 'liderfen' OR 'lidifen' OR 'liptan' OR 'lopane' OR 'los-u-fen' OR 'lotempen' OR 'malafene' OR 'maxagesic' OR 'mcn r 1451' OR 'medicol' OR 'medifen' OR 'medipren' OR 'mediprin' OR 'melfen' OR 'mensoton' OR 'metafen' OR 'midol 200' OR 'midol liquid gels' OR 'mig (drug)' OR 'mig forte' OR 'mig junior' OR 'mig pediatric' OR 'milifen' OR 'miofen' OR 'momenflogo' OR 'moment (ibuprofen)' OR 'momentact' OR 'momentact analgesico' OR 'momentfene' OR 'morrisons long lasting pain relief' OR 'motrin' OR 'motrin childrens' OR 'motrin ib' OR 'motrin migraine pain' OR 'mynosedin' OR 'nagifen-d' OR 'napacetin' OR 'neforan' OR 'neo borocillina infiammazione e dolore' OR 'neobrufen' OR 'neobrufen retard' OR 'neodol' OR 'neodol forte' OR 'neofen' OR 'neofen forte' OR 'neofen neo-forte' OR 'neofen plus' OR 'nerofen' OR 'neutropain' OR 'nobfelon' OR 'nobgen' OR 'norflam-t' OR 'noritis' OR 'norton' OR 'noubid' OR 'novidol' OR 'novogent' OR 'novogent n' OR 'novoprofen' OR 'nugin' OR 'nuprin' OR 'nureflex' OR 'nureflex lp' OR 'nurodon' OR 'nurofen' OR 'nurofen active' OR 'nurofen back pain' OR 'nurofen durance' OR 'nurofen expres' OR 'nurofen express' OR 'nurofen express femina' OR 'nurofen express forte' OR 'nurofen express maximum strength' OR 'nurofen express period pain' OR 'nurofen extra strength' OR 'nurofen fastine' OR 'nurofen febbre e dolore' OR 'nurofen febbre e dolore bambini' OR 'nurofen flextin' OR 'nurofen for children' OR 'nurofen for children cold, pain and fever' OR 'nurofen for children six plus' OR 'nurofen forte' OR 'nurofen forte express' OR 'nurofen gel' OR 'nurofen instant' OR 'nurofen joint and back pain relief max strength' OR 'nurofen joint and muscular pain relief' OR 'nurofen junior' OR 'nurofen junior fieber-und schmerzsaft' OR 'nurofen junior fiebersaft' OR 'nurofen long lasting' OR 'nurofen long lasting pain relief' OR 'nurofen max strength pain relief' OR 'nurofen musc' OR 'nurofen muscular pain relief' OR 'nurofen neo' OR 'nurofen neo femina' OR 'nurofen non-aqua' OR 'nurofen pain relief' OR 'nurofen pain relief max strength' OR 'nurofen patch' OR 'nurofen pediatrico' OR 'nurofen pleister' OR 'nurofen pour enfants' OR 'nurofen pre deti' OR 'nurofen pro deti' OR 'nurofen rapid' OR 'nurofen rapid forte' OR 'nurofen rapid relief maximum strength' OR 'nurofen recovery' OR 'nurofen voor kinderen' OR 'nurofen xpress' OR 'nurofen za djecu' OR 'nurofen zavance' OR 'nurofencaps' OR 'nurofenjunior' OR 'nurofenkid' OR 'nurofenkid febbre e dolore' OR 'nurofenplast' OR 'nurofentabs' OR 'nurofenteen' OR 'nuroflex' OR 'optalidon ibu' OR 'optifen' OR 'opturem' OR 'ostarin' OR 'ostofen' OR 'ozonol (ibuprofen)' OR 'paduden' OR 'paduden forte' OR 'paduden sr' OR 'paediatric advil' OR 'paidofebril' OR 'paidofen' OR 'panactive' OR 'panafen' OR 'pathofen' OR 'pedea' OR 'pediacare fever' OR 'pediatric advil' OR 'pediprofen' OR 'perdophen pediatrie' OR 'peribu' OR 'perofen' OR 'pfeil zahnschmerz forte' OR 'phorpain' OR 'phorpain gel' OR 'pirexin' OR 'potassium ibuprofen' OR 'proartinal' OR 'profen' OR 'profeno' OR 'proff' OR 'proff schmerzcreme' OR 'proflex' OR 'proris' OR 'provin' OR 'provon' OR 'quadrax' OR 'radian b' OR 'rafen' OR 'ranofen' OR 'rapidol s' OR 'rapidophen' OR 'rapidophen forte' OR 'ratiodolor' OR 'rd 13621' OR 'rd13621' OR 'rebugen' OR 'renidon' OR 'reuprofen' OR 'reuvol' OR 'rhelafen' OR 'rhelafen forte' OR 'riflonet' OR 'rofixdol febbre e dolore' OR 'roidenin' OR 'rolibfar' OR 'rufan' OR 'rufen' OR 'rupan' OR 'sainsbury`s healthcare long lasting pain relief' OR 'sakitol' OR 'saniprom' OR 'saridon n' OR 'sarixell' OR 'schmerz-dolgit' OR 'schufen' OR 'seclodin' OR 'sodium ibuprofen' OR 'solibu' OR 'solufen lidose' OR 'solvium' OR 'spalt' OR 'spalt forte' OR 'spalt migrane' OR 'spalt mobil' OR 'spididolpocket' OR 'stribu' OR 'subitene' OR 'superdrug children`s pain and fever relief' OR 'superdrug long lasting pain relief' OR 'syafen' OR 'syntofene' OR 'tab-profen' OR 'tabalon' OR 'tabalon 400' OR 'taskine' OR 'tatanal' OR 'tefin' OR 'tesco health long lasting pain relief' OR 'tofen' OR 'trendar' OR 'tricalma (ibuprofen)' OR 'trifene' OR 'tvingestin' OR 'u 18573' OR 'u18573' OR 'umafen' OR 'unipro' OR 'upfen' OR 'uprofen' OR 'urem' OR 'viamal febbre e dolore' OR 'wilko long lasting pain relief' OR 'winadol (ibuprofen)' OR 'zafen' OR 'zavryl' OR 'zenalgin' OR 'zentidol' OR 'zofen' OR 'ibuprofen' OR 'anticoagulant agent'/exp OR 'anti coagulant' OR 'anti coagulant agent' OR 'anti coagulant drug' OR 'anti coagulating agent' OR 'anticoagulant' OR 'anticoagulant drug' OR 'anticoagulants' OR 'anticoagulating agent' OR 'anticoagulation agent' OR 'anticoagulative agent' OR 'antithrombotic' OR 'antithrombotic agent' OR 'antithrombotic drug' OR 'oral anticoagulant' OR 'oral anticoagulant agent' OR 'anticoagulant agent' OR 'apixaban'/exp OR '1 (4 methoxyphenyl) 7 oxo 6 [4 (2 oxo 1 piperidinyl) phenyl] 1, 4, 5, 6, 7 pentahydropyrazolo [3, 4 c] pyridine 3 carboxamide' OR '1 (4 methoxyphenyl) 7 oxo 6 [4 (2 oxo 1 piperidinyl) phenyl] 4, 5, 6, 7 tetrahydropyrazolo [3, 4 c] pyridine 3 carboxamide' OR '1 (4 methoxyphenyl) 7 oxo 6 [4 (2 oxo 1 piperidinyl) phenyl] 4, 5, 6, 7 tetrahydro 1h pyrazolo [3, 4 c] pyridine 3 carboxamide' OR '1 (4 methoxyphenyl) 7 oxo 6 [4 (2 oxo 1 piperidyl) phenyl] 1, 4, 5, 6, 7 pentahydropyrazolo [3, 4 c] pyridine 3 carboxamide' OR '1 (4 methoxyphenyl) 7 oxo 6 [4 (2 oxo 1 piperidyl) phenyl] 4, 5, 6, 7 tetrahydropyrazolo [3, 4 c] pyridine 3 carboxamide' OR '1 (4 methoxyphenyl) 7 oxo 6 [4 (2 oxo 1 piperidyl) phenyl] 4, 5, 6, 7 tetrahydro 1h pyrazolo [3, 4 c] pyridine 3 carboxamide' OR '1 (4 methoxyphenyl) 7 oxo 6 [4 (2 oxopiperidin 1 yl) phenyl] 1, 4, 5, 6, 7 pentahydropyrazolo [3, 4 c] pyridine 3 carboxamide' OR '1 (4 methoxyphenyl) 7 oxo 6 [4 (2 oxopiperidin 1 yl) phenyl] 4, 5, 6, 7 tetrahydropyrazolo [3, 4 c] pyridine 3 carboxamide' OR '1 (4 methoxyphenyl) 7 oxo 6 [4 (2 oxopiperidin 1 yl) phenyl] 4, 5, 6, 7 tetrahydro 1h pyrazolo [3, 4 c] pyridine 3 carboxamide' OR '4, 5, 6, 7 tetrahydro 1 (4 methoxyphenyl) 7 oxo 6 [4 (2 oxo 1 piperidinyl) phenyl] 1 hydropyrazolo [3, 4 c] pyridine 3 carboxamide' OR '4, 5, 6, 7 tetrahydro 1 (4 methoxyphenyl) 7 oxo 6 [4 (2 oxo 1 piperidinyl) phenyl] pyrazolo [3, 4 c] pyridine 3 carboxamide' OR '4, 5, 6, 7 tetrahydro 1 (4 methoxyphenyl) 7 oxo 6 [4 (2 oxo 1 piperidyl) phenyl] 1 hydropyrazolo [3, 4 c] pyridine 3 carboxamide' OR '4, 5, 6, 7 tetrahydro 1 (4 methoxyphenyl) 7 oxo 6 [4 (2 oxopiperidin 1 yl) phenyl] 1 hydropyrazolo [3, 4 c] pyridine 3 carboxamide' OR '4, 5, 6, 7 tetrahydro 1 (4 methoxyphenyl) 7 oxo 6 [4 (2 oxopiperidin 1 yl) phenyl] pyrazolo [3, 4 c] pyridine 3 carboxamide' OR '4, 5, 6, 7 tetrahydro 1 (4 methoxyphenyl) 7 oxo 6 [4 (2 oxo 1 piperidinyl) phenyl] 1h pyrazolo [3, 4 c] pyridine 3 carboxamide' OR '4, 5, 6, 7 tetrahydro 1 (4 methoxyphenyl) 7 oxo 6 [4 (2 oxo 1 piperidyl) phenyl] 1h pyrazolo [3, 4 c] pyridine 3 carboxamide' OR '4, 5, 6, 7 tetrahydro 1 (4 methoxyphenyl) 7 oxo 6 [4 (2 oxopiperidin 1 yl) phenyl] 1h pyrazolo [3, 4 c] pyridine 3 carboxamide' OR 'aboxoma' OR 'apixaben' OR 'bms 562247' OR 'bms 562247 01' OR 'bms 562247-01' OR 'bms562247' OR 'bms562247 01' OR 'bms562247-01' OR 'eliques' OR 'eliquis' OR 'lunast' OR 'pf 0465257' OR 'pf0465257' OR 'tah 3311' OR 'tah 3341' OR 'tah3311' OR 'tah3341' OR 'apixaban' OR 'rivaroxaban'/exp OR '5 chloro n [ [2 oxo 3 [4 (3 oxo 4 morpholinyl) phenyl] 1, 3 oxazolidin 5 yl] methyl] 2 thiophenecarboxamide' OR '5 chloro n [ [2 oxo 3 [4 (3 oxo 4 morpholinyl) phenyl] 5 oxazolidinyl] methyl] 2 thiophenecarboxamide' OR '5 chloro n [ [2 oxo 3 [4 (3 oxo 4 morpholyl) phenyl] 1, 3 oxazolidin 5 yl] methyl] 2 thiophenecarboxamide' OR '5 chloro n [ [2 oxo 3 [4 (3 oxo 4 morpholyl) phenyl] 5 oxazolidyl] methyl] 2 thiophenecarboxamide' OR '5 chloro n [ [2 oxo 3 [4 (3 oxomorpholin 4 yl) phenyl] 1, 3 oxazolidin 5 yl] methyl] thiophene 2 carboxamide' OR '5 chloro n [ [2 oxo 3 [4 (3 oxomorpholin 4 yl) phenyl] oxazolidin 5 yl] methyl] thiophene 2 carboxamide' OR '5 chloro n [ [2 oxo 3 [4 (3 oxomorpholino) phenyl] 5 oxazolidinyl] methyl] 2 thiophenecarboxamide' OR 'aban (drug)' OR 'assubex' OR 'ast 8294' OR 'ast8294' OR 'bay 59 7939' OR 'bay 59-7939' OR 'bay 597939' OR 'bay59 7939' OR 'bay59-7939' OR 'bay597939' OR 'bs 112' OR 'bs112' OR 'dst 8294' OR 'dst8294' OR 'jnj 39039039' OR 'jnj39039039' OR 'kriva' OR 'naxat' OR 'rivaro' OR 'rivarolto' OR 'rivaxa' OR 'throsaben' OR 'xanirva' OR 'xarelto' OR 'xerdoxo' OR 'xindus' OR 'rivaroxaban' OR 'edoxaban'/exp OR 'du 176' OR 'du 176b' OR 'du176' OR 'du176b' OR 'edoxaban tosilate' OR 'edoxaban tosilate hydrate' OR 'edoxaban tosylate' OR 'edoxaban tosylate hydrate' OR 'endoxaban' OR 'lixiana' OR 'n (5 chloro 2 pyridinyl) n` [4 (n, n dimethylcarbamoyl) 2 (5 methyl 4, 5, 6, 7 tetrahydrothiazolo [5, 4 c] pyridine 2 carboxamido) cyclohexyl] oxamide' OR 'roteas' OR 'savaysa' OR 'edoxaban' OR 'fondaparinux'/exp OR 'arixtra' OR 'fondaparin' OR 'fondaparin sodium' OR 'fondaparinux sodium' OR 'gsk 576428' OR 'gsk576428' OR 'ic 851589' OR 'ic851589' OR 'org 31540' OR 'org31540' OR 'quixidar' OR 'sr 90107' OR 'sr 90107a' OR 'sr90107' OR 'sr90107a' OR 'xantidar' OR 'fondaparinux' OR 'heparin'/exp OR 'alpha heparin' OR 'ammonium heparinate' OR 'benzalkonium heparin' OR 'beparine' OR 'clarin' OR 'contusol' OR 'disebrin' OR 'eleparon' OR 'elheparin' OR 'elheparon' OR 'endogenous heparin' OR 'epiheparin' OR 'gag 98' OR 'helberina' OR 'hep flush kit' OR 'hep lock' OR 'hep-lock' OR 'hep-lock u/p' OR 'hep-pak cvc' OR 'hepaflex' OR 'hepalean' OR 'heparin injection b.p.' OR 'heparin leo' OR 'heparin lock flush' OR 'heparin lock flush plus sodium chloride' OR 'heparin lock flush preservative free' OR 'heparin monosulfate' OR 'heparin monosulphate' OR 'heparin novo' OR 'heparin ointment' OR 'heparin potassium' OR 'heparin sodium' OR 'heparin sodium 1, 000 units and sodium chloride 0.9%' OR 'heparin sodium 1, 000 units in dextrose 5%' OR 'heparin sodium 1, 000 units in sodium chloride 0.9%' OR 'heparin sodium 10, 000 units in dextrose 5%' OR 'heparin sodium 10, 000 units in sodium chloride 0.45%' OR 'heparin sodium 10, 000 units in sodium chloride 0.9%' OR 'heparin sodium 12, 500 units in dextrose 5%' OR 'heparin sodium 12, 500 units in sodium chloride 0.45%' OR 'heparin sodium 12, 500 units in sodium chloride 0.9%' OR 'heparin sodium 2, 000 units and sodium chloride 0.9%' OR 'heparin sodium 2, 000 units in dextrose 5%' OR 'heparin sodium 2, 000 units in sodium chloride 0.9%' OR 'heparin sodium 20, 000 units and dextrose 5%' OR 'heparin sodium 20, 000 units in dextrose 5%' OR 'heparin sodium 25, 000 units and dextrose 5%' OR 'heparin sodium 25, 000 units in dextrose 5%' OR 'heparin sodium 25, 000 units in sodium chloride 0.45%' OR 'heparin sodium 25, 000 units in sodium chloride 0.9%' OR 'heparin sodium 5, 000 units and sodium chloride 0.9%' OR 'heparin sodium 5, 000 units in dextrose 5%' OR 'heparin sodium 5, 000 units in sodium chloride 0.45%' OR 'heparin sodium 5, 000 units in sodium chloride 0.9%' OR 'heparin sodium b braun' OR 'heparin sodium preservative free' OR 'heparin subcutaneous' OR 'heparin sulfate' OR 'heparin sulfuric acid' OR 'heparin sulphate' OR 'heparina' OR 'heparina leo' OR 'heparinate sodium' OR 'heparine' OR 'heparine choay' OR 'heparine novo' OR 'heparinic acid' OR 'heparitin monosulfate' OR 'heparitin monosulphate' OR 'hepcon' OR 'hepflush-10' OR 'hepsal' OR 'inhepar' OR 'inviclot' OR 'lipo hepin' OR 'lipo-hepin' OR 'lipohepin' OR 'liquaemin' OR 'liquaemin lock flush' OR 'liquaemin sodium' OR 'liquaemin sodium preservative free' OR 'liquemin' OR 'liquemine' OR 'menaven' OR 'monoparin' OR 'mucoitin polysulfate' OR 'mucoitin polysulfate ester' OR 'mucoitin polysulphate' OR 'mucoitin polysulphate ester' OR 'mucoitin sodium polysulfate' OR 'mucoitin sodium polysulphate' OR 'multiparin' OR 'nevparin' OR 'noparin' OR 'panheparin' OR 'panhepin' OR 'panheprin' OR 'parinix' OR 'phlebotroy qps' OR 'praecivenin' OR 'pularin' OR 'sodium heparin' OR 'thrombareduct' OR 'thrombo vetren' OR 'thromboliquin' OR 'thromboliquine' OR 'thrombophlogat' OR 'thrombophob' OR 'thrombophob gel' OR 'thromboreduct' OR 'thrombosamine' OR 'thrombosamine heparin' OR 'thrombosamine heparine' OR 'unfractionated heparin' OR 'uniparin' OR 'vetren' OR 'vister' OR 'vr 496' OR 'vr496' OR 'heparin' OR 'low molecular weight heparin'/exp OR 'bm 2123' OR 'bm2123' OR 'choay' OR 'depolymerized heparin' OR 'ebpm 1' OR 'ebpm 2' OR 'ebpm 3' OR 'ebpm1' OR 'ebpm2' OR 'ebpm3' OR 'ff 1034' OR 'ff1034' OR 'fr 860' OR 'fr860' OR 'gag 869' OR 'heparin lmw 2133' OR 'heparin, low molecular weight' OR 'heparin, low-molecular-weight' OR 'heparin, low molecular weight fraction' OR 'low molecular heparin' OR 'nm heparin' OR 'pk 007' OR 'sandoz 5100' OR 'sandoz 6700' OR 'traxyparine' OR 'low molecular weight heparin' OR 'dalteparin'/exp OR 'dalteparin sodium' OR 'fragmin' OR 'fragmin p forte' OR 'fragmine' OR 'k 2165' OR 'k2165' OR 'kabi 2165' OR 'low liquemin' OR 'dalteparin' OR 'enoxaparin'/exp OR 'arovi' OR 'clexan' OR 'clexane' OR 'clexane 40' OR 'clexane forte' OR 'clexane multidose' OR 'clexane t' OR 'colevance' OR 'crusia' OR 'decipar' OR 'enoxaparin sodium' OR 'ghemaxan' OR 'hepaxane' OR 'inhixa' OR 'klexane' OR 'ledraxen' OR 'losima' OR 'lovenox' OR 'lovenox (preservative free)' OR 'neoparin' OR 'neoparin-nx' OR 'percolozin' OR 'pk 10169' OR 'pk10169' OR 'qualiop klinik' OR 'rovinadil' OR 'rp 54563' OR 'rp54563' OR 'thorinane' OR 'enoxaparin' OR 'argatroban'/exp OR '1 [5 [ (aminoiminomethyl) amino] 1 oxo 2 [ [(1, 2, 3, 4 tetrahydro 3 methyl 8 quinolinyl) sulfonyl] amino] pentyl] 4 methyl 2 piperidinecarboxylic acid' OR '1 [5 [ (aminoiminomethyl) amino] 1 oxo 2 [ [(1, 2, 3, 4 tetrahydro 3 methylquinolin 8 yl) sulfonyl] amino] pentyl] 4 methylpiperidine 2 carboxylic acid' OR '1 [n (1, 2, 3, 4 tetrahydro 3 methyl 8 quinolinesulfonyl) arginyl] 4 methylpipecolic acid' OR '1 [n (1, 2, 3, 4 tetrahydro 3 methylquinoline 8 sulfonyl) arginyl] 4 methylpipecolic acid' OR '4 methyl 1 [n alpha (3 methyl 1, 2, 3, 4 tetrahydroquinoline 8 sulfonyl) arginyl] piperidine 2 carboxylic acid' OR '4 methyl 1 [n2 (1, 2, 3, 4 tetrahydro 3 methyl 8 quinolylsulfonyl) arginyl] pipecolic acid' OR '4 methyl 1 [n2 (1, 2, 3, 4 tetrahydro 3 methylquinolyl 8 sulfonyl) arginyl] pipecolic acid' OR '4 methyl 1 [n2 (3 methyl 1, 2, 3, 4 tetrahydro 8 quinolinesulfonyl) arginyl] 2 piperidinecarboxylic acid' OR '4 methyl 1 [n2 (3 methyl 1, 2, 3, 4 tetrahydroquinoline 8 sulfonyl) arginyl] piperidine 2 carboxylic acid' OR '4 methyl 1 [nalpha (3 methyl 1, 2, 3, 4 tetrahydro 8 quinolinesulfonyl) arginyl] 2 piperidinecarboxylic acid' OR 'acova' OR 'arganova' OR 'argatra' OR 'argatroban in 0.9% sodium chloride' OR 'argatroban in dextrose' OR 'argatroban in sodium chloride' OR 'argatroban in sodium chloride 0.9%' OR 'argatroban monohydrate' OR 'argipidine' OR 'dk 7419' OR 'dk7419' OR 'exembol' OR 'gn 1600' OR 'gn1600' OR 'mci 9038' OR 'mci9038' OR 'md 805' OR 'md805' OR 'novastan' OR 'om 805' OR 'om805' OR 'slonnon' OR 'argatroban' OR 'angiomax' OR 'angiomax rtu' OR 'angiox' OR 'bg 8967' OR 'bg8967' OR 'bivalirudin in sodium chloride 0.9%' OR 'bivalurudin trifluoroacetate' OR 'd phe pro arg pro (gly) 4 desulfohirudin [53-64]' OR 'd phe pro arg pro gly gly gly gly asn gly asp phe glu glu ile pro glu glu tyr leu' OR 'dextro phenylalanylprolylarginylprolylglycylglycylglycylglycylasparaginylglycyl alpha aspartylphenylalanyl alpha glutamyl alpha glutamylisoleucylprolyl alpha glutamyl alpha glutamyl alpha tyrosyleucine' OR 'dextro phenylalanylprolylarginylprolylglycylglycylglycylglycylasparaginylglycyl alpha aspartylphenylalanyl alpha glutamyl alpha glutamylisoleucylprolyl alpha glutamyl alpha glutamyl alpha tyrosyleucine bis (2, 2, 2 trifluoroacetate)' OR 'ep 6101' OR 'ep6101' OR 'hirulog' OR 'hirulog 1' OR 'bivalirudin' OR 'desulfatohirudin'/exp OR 'cgp 39393' OR 'cgp39393' OR 'desirudin' OR 'desirudin recombinant' OR 'hirudin [63 tyrosine]' OR 'ipravask' OR 'iprivask' OR 'revasc' OR 'desulfatohirudin' OR 'warfarin'/exp OR '1 (4` hydroxy 3` coumarinyl) 1 phenyl 3 butanone' OR '3 acetonylbenzonyl 4 hydroxy coumarinedimethylaminoethanol' OR '3 alpha phenyl beta acetylethyl 4 hydroxycoumarin' OR '3 (alpha acetonylbenzyl) 4 hydroxycoumarin' OR '4 hydroxy 3 (3 oxo 1 phenylbutyl) 2h chromen 2 one' OR 'acetonylbenzylhydroxycoumarin' OR 'adoisine' OR 'aldocumar' OR 'alpha acetonylbenzyl 4 hydroxycoumarin dimethylaminoethanol' OR 'antrombin k' OR 'athrombin' OR 'athrombin k' OR 'athrombin-k' OR 'athrombine k' OR 'athrombinek' OR 'befarin' OR 'bms 565793' OR 'bms565793' OR 'carfin' OR 'circuvit' OR 'compound 42' OR 'coumadan' OR 'coumadan sodico' OR 'coumadin' OR 'coumadin sodium' OR 'coumadine' OR 'coumafene' OR 'coumaphene' OR 'd warfarin' OR 'dagonal' OR 'dextro warfarin' OR 'farin' OR 'jantoven' OR 'kumatox' OR 'l warfarin' OR 'levo warfarin' OR 'maforan' OR 'marevan' OR 'marevan forte' OR 'marfarin' OR 'martefarin' OR 'orfarin' OR 'panwarfarin' OR 'panwarfin' OR 'potassium warfarin' OR 'prothromadin' OR 'r warfarin' OR 'simarc-2' OR 'sodium warfarin' OR 'sodium warfarinum' OR 'sofarin' OR 'tintorane' OR 'uniwarfin' OR 'wafarin' OR 'waran' OR 'warf compound 42' OR 'warfant' OR 'warfar' OR 'warfarin 2 (dimethylamino) ethanol' OR 'warfarin potassium' OR 'warfarin sodium' OR 'warfarine' OR 'warfarinum sodium' OR 'warfil 5' OR 'warfilone' OR 'warfin' OR 'warnerin' OR 'warfarin' OR '30 ethyl 33 (1 hydroxy 2 methyl 4 hexen 1 yl) 6, 9, 18, 24 tetraisobutyl 3, 21 diisopropyl 1, 4, 7, 10, 12, 15, 19, 25, 28 nonamethyl 1, 4, 7, 10, 13, 16, 19, 22, 25, 28, 31 undecaazacyclotritriacontane 2, 5, 8, 11, 14, 17, 20, 23, 26, 29, 32 undecone' OR 'adi 628' OR 'adi628' OR 'atopica' OR 'capimune' OR 'capsorin' OR 'cequa' OR 'cgc 1072' OR 'cgc1072' OR 'ciclograft' OR 'ciclomulsion' OR 'cicloral' OR 'ciclosporin' OR 'ciclosporin a' OR 'ciclosporine' OR 'cipol' OR 'cipol-n' OR 'ciqorin' OR 'consupren' OR 'cyclaid' OR 'cyclasol' OR 'cyclic [alanyl dextro alanyl n methylleucyl n methylleucyl n methylvalyl (6, 7 didehydro 3 hydroxy n, 4 dimethyl 2 aminooctanoyl) 2 aminobutanoyl n methylglycyl n methylleucylvalyl n methylleucyl]' OR 'cyclo [[3 hydroxy 4 methyl 2 (methylamino) 6 octenoyl] 2 aminobutyryl n methylglycyl n methylleucylvalyl n methylleucylalanyl dextro alanyl n methylleucyl n methylleucyl n methylvalyl]' OR 'cyclo [alanyl dextro alanyl n methylleucyl n methylleucyl n methylvalyl (6, 7 didehydro 3 hydroxy n, 4 dimethyl 2 aminooctanoyl) 2 aminobutanoyl n methylglycyl n methylleucylvalyl n methylleucyl]' OR 'cyclokat' OR 'cyclosporin' OR 'cyclosporin a' OR 'cyclosporin neoral' OR 'cyclosporine a' OR 'de 076' OR 'de076' OR 'deximune' OR 'equoral' OR 'gengraf' OR 'ikervis' OR 'iminoral' OR 'immunosporin' OR 'implanta' OR 'imunofar' OR 'imusporin' OR 'lx 201' OR 'lx201' OR 'mc2 03' OR 'mc203' OR 'menelri' OR 'mitogard' OR 'mtd 202' OR 'mtd202' OR 'neciclopin' OR 'neoimmun' OR 'neoplanta (drug)' OR 'neoral' OR 'neoral-sandimmun' OR 'neuro-stat (drug)' OR 'neurostat (drug)' OR 'nm 0133' OR 'nm 133' OR 'nm0133' OR 'nm133' OR 'nova 22007' OR 'nova22007' OR 'ol 27400' OR 'ol27400' OR 'olo 400' OR 'olo500' OR 'opph 088' OR 'opph088' OR 'opsisporin' OR 'optimmune' OR 'otx 101' OR 'otx101' OR 'p 3072' OR 'p3072' OR 'padciclo' OR 'papilock' OR 'pulminiq' OR 'ramihyphin a' OR 'restasis' OR 'restaysis' OR 'sanciclo' OR 'sandimmun' OR 'sandimmun neoral' OR 'sandimmun optoral' OR 'sandimmune' OR 'sandimmune neoral' OR 'sandimun' OR 'sandimun neoral' OR 'sandimune' OR 'sang 35' OR 'sang35' OR 'sangcya' OR 'seciera' OR 'sp 14019' OR 'sp14019' OR 'sporilen' OR 'sti 0529' OR 'sti0529' OR 't 1580' OR 't1580' OR 'vanquoral' OR 'vekacia' OR 'verkazia' OR 'vevizye' OR 'vevye' OR 'zinograf me' OR 'cyclosporine' OR '7 chloro 4 [4 [ethyl (2 hydroxyethyl) amino] 1 methylbutylamino] quinoline' OR '7 chloro 4 [4 [ethyl (2 hydroxyethyl) amino] 1 methylbutylamino] quinoline diphosphate' OR 'apo-hydroxychloroquine' OR 'chloroquinol' OR 'ercoquin' OR 'hydrochloroquine' OR 'hydrocloroquine' OR 'hydroxychloroquine liposome' OR 'oxychloroquine' OR 'quensyl' OR 'sn 8137' OR 'sn8137' OR 'tlc 19' OR 'tlc19' OR 'win 1258' OR 'win1258' OR 'hydroxychloroquine' OR 'tacrolimus'/exp OR 'adoport' OR 'advagraf' OR 'ascezo' OR 'astagraf' OR 'astagraf xl' OR 'capexion' OR 'cellmune (tacrolimus)' OR 'cidimus' OR 'conferoport' OR 'crilomus' OR 'dailiport' OR 'dermitopic' OR 'envarsus' OR 'envarsus xr' OR 'fk 506' OR 'fk-506' OR 'fk506' OR 'fr 900506' OR 'fr900506' OR 'fujimycin' OR 'gecrol' OR 'graceptor' OR 'hecoria' OR 'l 679934' OR 'l679934' OR 'mld 987' OR 'mld987' OR 'modigraf' OR 'mr 4' OR 'mr4' OR 'mtd 219' OR 'mtd219' OR 'mustopic oint' OR 'prograf' OR 'prograft' OR 'protopic' OR 'protopic ointment' OR 'protopy' OR 'rtu 007' OR 'rtu007' OR 'tac-lac (drug)' OR 'tacforius' OR 'tacni' OR 'tacro-cell' OR 'tacrocel' OR 'tacrolimus hydrate' OR 'tacrosolv' OR 'takrozem' OR 'talymus' OR 'tsukubaenolide' OR 'tacrolimus' OR 'sirolimus'/exp OR '1, 18 dihydroxy 12 [1 (4 hydroxy 3 methoxycyclohexyl) 2 propanyl] 19, 30 dimethoxy 15, 17, 21, 23, 29, 35 hexamethyl 11, 36 dioxa 4 azatricyclo [30.3.1.0 (4, 9)] hexatriaconta 16, 24, 26, 28 tetraene 2, 3, 10, 14, 20 pentone' OR '1, 18 dihydroxy 12 [1 (4 hydroxy 3 methoxycyclohexyl) 2 propyl] 19, 30 dimethoxy 15, 17, 21, 23, 29, 35 hexamethyl 11, 36 dioxa 4 azatricyclo [30.3.1.0 (4, 9)] hexatriaconta 16, 24, 26, 28 tetraene 2, 3, 10, 14, 20 pentone' OR '1, 18 dihydroxy 12 [1 (4 hydroxy 3 methoxycyclohexyl) isopropyl] 19, 30 dimethoxy 15, 17, 21, 23, 29, 35 hexamethyl 11, 36 dioxa 4 azatricyclo [30.3.1.0 (4, 9)] hexatriaconta 16, 24, 26, 28 tetraene 2, 3, 10, 14, 20 pentone' OR '1, 18 dihydroxy 12 [1 (4 hydroxy 3 methoxycyclohexyl) propan 2 yl] 19, 30 dimethoxy 15, 17, 21, 23, 29, 35 hexamethyl 11, 36 dioxa 4 azatricyclo [30.3.1.0 (4, 9)] hexatriaconta 16, 24, 26, 28 tetraene 2, 3, 10, 14, 20 pentone' OR 'abi 009' OR 'abi009' OR 'albumin bound rapamycin' OR 'ay 22989' OR 'ay22989' OR 'coll-r' OR 'cypher (drug)' OR 'de 109' OR 'de109' OR 'drgt18 2' OR 'drgt182' OR 'fyarro' OR 'hyftor' OR 'ms r001' OR 'msr001' OR 'nab-rapamycin' OR 'nab-sirolimus' OR 'npc 12' OR 'npc 12g' OR 'npc 12t' OR 'npc 12y' OR 'npc12' OR 'npc12g' OR 'npc12t' OR 'npc12y' OR 'opsiria' OR 'pascomer' OR 'perceiva' OR 'ptx 001' OR 'ptx 367' OR 'ptx001' OR 'ptx367' OR 'rapalimus' OR 'rapammune' OR 'rapamune' OR 'rapamycin' OR 'sila 9268a' OR 'sila9268a' OR 'sirogen' OR 'stn 10109' OR 'stn 1010900' OR 'stn 1010905' OR 'stn10109' OR 'stn1010900' OR 'stn1010905' OR 'tarzifyx' OR 'tavt 18' OR 'tavt18' OR 'tmb 002' OR 'tmb002' OR 'wy 090217' OR 'wy090217' OR 'sirolimus' OR 'leukotriene b4 receptor antagonist' OR '5, 12 dihydroxy 6, 8, 10, 14 eicosatetraenoic acid' OR '5, 12 dihydroxy 6, 8, 10, 14 icosatetraenoic acid' OR '6, 8, 10, 14 icosatetraenoic acid, 5, 12 dihydroxy' OR 'leukotriene b4' OR 'leukotriene b4 inhibitor' OR 'leukotriene b-4' OR 'leukotriene b 4' OR 'platelet rich plasma' OR 'platelet-rich plasma' OR 'thrombocyte rich plasma' OR 'immunosuppressive agent'/exp OR 'drug, immunosuppressive' OR 'immune suppressant' OR 'immuno suppressive drug' OR 'immunodepressant' OR 'immunodepressant agent' OR 'immunosuppressant' OR 'immunosuppressant agent' OR 'immunosuppressant drug' OR 'immunosuppressive agents' OR 'immunosuppressive drug' OR 'immunosuppressive substance' OR 'immunosuppressives' OR 'immunosuppressor' OR 'immunosuppressive agent' OR 'immunosuppressants') AND ('breast cancer lymphedema' OR 'breast cancer lymphoedema' OR 'breast cancer-related lymphoedema' OR 'post-breast cancer lymphedema' OR 'post-breast cancer lymphoedema' OR 'breast cancer-related lymphedema' OR 'breast cancer lymphedemas' OR 'breast cancer lymphoedemas' OR 'breast cancer-related lymphoedemas' OR 'post-breast cancer lymphedemas' OR 'post-breast cancer lymphoedemas' OR 'breast cancer-related lymphedemas' OR 'breast cancer treatment-related lymphedema' OR 'breast cancer-related arm lymphedema' OR 'breast cancer treatment-related lymphedemas' OR 'breast cancer-related arm lymphedemas' OR 'postmastectomy lymphedema' OR 'postmastectomy lymphedemas' OR 'post-mastectomy lymphedema' OR 'post-mastectomy lymphedemas' OR 'post-mastectomy lymphoedema') | | | | |
| **2D.** Cumulative Index to Nursing and Allied Health Literature search strategy | | | | |
| (MH "Aspirin" OR 2-(Acetyloxy)benzoic Acid OR Acetylsalicylic Acid OR Solprin OR Polopirin OR Solupsan OR Easprin OR Zorprin OR Acetysal OR Acylpyrin OR Colfarit OR Ecotrin OR Endosprin OR Magnecyl OR Micristin OR Polopiryna OR Aloxiprimum OR Dispril) OR MH "Antiinflammatory Agents, Non-Steroidal" OR Non-Steroidal Anti-Inflammatory Agent OR Non Steroidal Anti Inflammatory Agent OR NSAID OR NSAIDs OR Nonsteroidal Anti-Inflammatory Agent OR Nonsteroidal Anti Inflammatory Agent OR Nonsteroidal Antiinflammatory Agents OR Non-Steroidal Anti-Inflammatory Agents OR Non Steroidal Anti Inflammatory Agents OR Nonsteroidal Anti-Inflammatory Agents OR Nonsteroidal Anti Inflammatory Agents OR Anti-Inflammatory Analgesics OR Aspirin-Like Agents OR Aspirin Like Agents OR Aspirin-Like Agent OR Aspirin Like Agent) OR "Ketoprofen" OR "2-(3-Benzoylphenyl)propionic Acid OR Benzoylhydratropic Acid" OR Alrheumum OR Orudis OR Profenid OR "19,583 RP" OR "RP, 19,583" OR "RP-19583" OR "RP19583" OR "RP 19583" OR Alrheumat) OR MH "Ibuprofen" OR ibuprofen OR "alpha-Methyl-4-(2-methylpropyl)benzeneacetic Acid OR Benzeneacetic Acid, alpha-methyl-4-(2-methylpropyl)- trimethylsilyl ester" OR Ibuprofen-Zinc OR Ibuprofen Zinc OR Salprofen OR Brufen OR Motrin OR Ibumetin OR Nuprin OR Rufen OR Trauma-Dolgit Gel OR Trauma Dolgit Gel OR Advil) OR MH "Anticoagulants+" OR Anticoagulant Drug OR Anticoagulant Agent OR Anticoagulation Agents OR Anticoagulant Drugs OR Anticoagulant Agents OR Anticoagulant OR Indirect Thrombin Inhibitors) OR “apixaban” OR apixaban OR Eliquis OR "BMS 562247" OR "BMS-562247" OR "BMS562247" OR "BMS-562247-01") OR "Rivaroxaban” OR "BAY 59 7939" OR Xarelto OR "5-chloro-N-(((5S)-2-oxo-3-(4-(3-oxomorpholin-4-yl)phenyl)-1,3-oxazolidin-5-yl)methyl)thiophene-2-carboxamide" OR "BAY 59-7939" OR "BAY 597939") OR Edoxaban OR “edoxaban” OR "N-(5-chloropyridin-2-yl)-N'-((1S,2R,4S)-4-(N,N-dimethylcarbamoyl)-2-(5-methyl-4,5,6,7- tetrahydro(1,3)thiazolo(5,4-c)pyridine-2-carboxamido)cyclohexyl)oxamide" OR "DU-176b" OR "DU-176" OR edoxaban tosylate OR "N-(5-chloropyridin-2-yl)-N'-((1S,2R,4S)-4-(N,N-dimethylcarbamoyl)-2-(5-methyl-4,5,6,7-tetrahydrothiazolo(5,4-c)pyridine-2-carboxamido)cyclohexyl)ethanediamide p-toluenesulfonate monohydrate" OR Savaysa) OR “Fondaparinux” OR Fondaparinux OR Fondaparinux Sodium OR Arixtra OR Quixidar) OR MH "Heparin+" OR Heparin OR Low Molecular Weight Heparin OR Low-Molecular Weight Heparin OR LMWH OR Heparinic Acid OR Unfractionated Heparin OR Sodium Heparin OR Heparin Sodium OR alpha-Heparin OR alpha Heparin OR Liquaemin) OR MH "Dalteparin Sodium" OR Dalteparin OR Tedelparin OR Dalteparin Sodium OR Fragmin OR Fragmine OR "Kabi-2165" OR "Kabi2165" OR "Kabi 2165" OR "FR-860" OR "FR860" OR "FR 860") OR MH "Heparin, Low-Molecular-Weight" OR Enoxaparin OR Enoxaparine OR "EMT-966" OR "EMT966" OR "EMT 966" OR "EMT-967" OR "EMT967" OR "EMT 967" OR Clexane OR "PK-10,169" OR "PK 10,169" OR "PK10,169" OR "PK-10169" OR "PK10169" OR "PK 10169" OR Lovenox) OR MH "Argatroban" OR Argatroban OR "2R,4R)-1-(5-((aminoiminomethyl)amino)-1-oxo-2-(((1,2,3,4-tetrahydro-3-methyl-8-quinolinyl)sulfonyl)amino)pentyl)-4-methyl-2-piperidinecarboxylic acid monohydrate OR MMTQAP OR MPQA" OR argatroban monohydrate OR argatroban hydrate OR "MD 805" OR "MD-805" OR "MD805" OR Acova OR Novastan OR argatroban anhydrous OR "(2R,4R)-1-(5-((aminoiminomethyl)amino)-1-oxo-2-(((1,2,3,4-tetrahydro-3-methyl-8-quinolinyl)sulfonyl)amino)pentyl)-4-methyl-2-piperidinecarboxylic acid" OR "(21R)-argatroban anhydrous" OR "(2R,4R)-1-((2S)-5-((aminoiminomethyl)amino)-1-oxo-2-((((3R)-1,2,3,4-tetrahydro-3-methyl-8-quinolinyl)sulfonyl)amino)pentyl)-4-methyl-2-piperidinecarboxylic acid" OR "(21S)-argatroban anhydrous PR (2R,4R)-1-((2S)-5-((aminoiminomethyl)amino)-1-oxo-2-((((3S)-1,2,3,4-tetrahydro-3-methyl-8-quinolinyl)sulfonyl)amino)pentyl)-4-methyl-2-piperidinecarboxylic acid" OR "(21S)-argatroban" OR "MCI 9038" OR "MCI-9038" OR (21R)-argatroban) OR MH "Bivalirudin" OR bivalirudin OR "L-leucine, D-phenylalanyl-L-prolyl-L-arginyl-L-prolylglycylglycylglycylglycyl-L-asparaginylglycyl-L-alpha-aspartyl-L-phenylalanyl-L-alpha-glutamyl-L-alpha-glutamyl-L-isoleucyl-L-prolyl-L-alpha-glutamyl-L-alpha-glutamyl-L-tyrosyl- OR Phe-Pro-Arg-Pro-(Gly)4 desulfato-Tyr63'-hirugen" OR "Phe-Pro-Arg-Pro-(Gly)4-Asn-Gly-Asp-Phe-Glu-Glu-Ile-Pro-Glu-Glu-Tyr-Leu OR Phe-Pro-Arg-Pro-(Gly)4-desulfohirudin-(53-64)" OR "BG 8967" OR "BG-8967" OR "BG8967" OR Hirulog OR Hirulog-1 OR Angiomax OR Angiomax RTU OR "CTB-001" OR bivalirudin trifluoroacetate) OR MH "Dabigatran Etexilate" OR Dabigatran OR "N-((2-(((4-(aminoiminomethyl)phenyl)amino)methyl)-1-methyl-1H-benzimidazol-5-yl)carbonyl)-N-2-pyridinyl-beta-alanine" OR Dabigatran Etexilate OR "BIBR 1048" OR Pradaxa OR Dabigatran Etexilate Mesylate) OR “Desirudin” OR Desirudin OR desulfatohirudin OR desulphatohirudin OR recombinant HV1 hirudin OR "CGP 39393" OR "CGP-39393" OR "IK-HIR02" OR Revasc OR Iprivask) OR MH "Warfarin" OR Warfarin OR Coumadin OR "4-Hydroxy-3-(3-oxo-1-phenylbutyl)-2H-1-benzopyran-2-one" OR Warfarin Potassium OR Warfarin Sodium OR Marevan OR Gen-Warfarin OR Aldocumar OR Coumadine OR Apo-Warfarin OR Tedicumar OR Warfant) OR MH "Cyclosporine" OR Cyclosporin OR Ciclosporin OR Cyclosporin OR Cyclosporin A OR Cyclosporine A OR CsA-Neoral OR CsANeoral OR CsA Neoral OR Sandimmun Neoral OR CyA-NOF OR CyA NOF OR Sandimmune OR Sandimmun OR Neoral OR "OL 27-400" OR "OL 27400" OR "OL 27 400") OR MH "Hydroxychloroquine" OR hydroxychloroquine OR Hydroxychlorochin OR Oxychloroquine OR Oxychlorochin OR Plaquenil OR Hydroxychloroquine Sulfate OR Hydroxychloroquine Sulfate (1:1) Salt) OR (MH "Tacrolimus" OR Tacrolimus OR Prograft OR Anhydrous Tacrolimus OR "FK-506" OR "FK 506" OR "FK506" OR "FR-900506" OR "FR900506" OR "FR 900506") OR MH "Sirolimus" OR Sirolimus OR Rapamycin OR Rapamune OR "AY 22-989" OR "AY 22989" OR "AY 22 989" OR "I-2190A" OR "I2190A" OR "I 2190A") OR (Leukotriene B4 antagonist OR Leukotriene B4 inhibitor) OR “Leukotriene B4” OR MH "Leukotriene Antagonists" OR Leukotriene B4 OR Leukotriene B-4 OR "Leukotriene B 4" OR LTB4 OR "5,12-diHETE" OR "5,12 diHETE" OR "5,12-HETE" OR "5,12 HETE" OR "Leukotrienes B" OR "Leukotriene B") OR MH "Platelet-Rich Plasma+" OR Platelet Rich Plasma OR Platelet-Rich Plasma) OR MH "Immunosuppressive Agents+" OR Immunosuppressive Agent OR Immunosuppressant OR Immunosuppressants) OR (56 BaP OR benzopyrone OR coumarin OR cyclo 3 fort OR "cyclo 3 fort" OR hesperidin methylchalcone OR ruscus aculeatus OR butcher's-broom OR daflon OR diosmin OR diosmetin 7-o-rutinoside OR hesperidin OR "BN 165" OR ginkgo biloba OR troxerutin OR foeniculum vulgare OR fennel OR salvia officinalis OR sage OR geranium OR juniper OR black pepper OR piper nigrum OR oak OR quercus robur OR robuvit OR kampo OR goreisan OR goshajinkigan OR linfadren OR arbutin OR weiyang huoxue washin OR zingiber officinale rosc OR cinnamomum OR zanthoxylum OR angelica sinensis OR sairei to OR chai-ling-tang OR bupleurum root OR pinellia tuber OR alisma rhizome OR scutellaria root OR ginseng OR poria sclerotium OR polyporus sclerotium OR atractylodes lancea rhizome OR jujube OR glycyrrhiza OR licorice OR cinnamon OR saikosaponin OR lypmhomyosot OR gentiana lutea OR pinus sylvestris OR scrophularia nodosa OR equisetum hyemale OR fumaria officianlis OR geranium robertianum OR nasturtium officinale OR sarsaparilla OR myositis arvensis OR teucrium scorodonia OR veronica officianalis OR sulfuretin OR rhus verniciflua stokes OR butein OR juzentaihoto OR tara spinosa OR caesalpinia spinosa OR achillea millefolium OR centella asiatica OR olivari OR olive OR olea europaea OR "3,4-dihydroxyphenyl ethanol" OR hydroxytyrosol OR wu ling san OR rhizoma alismatis OR polyporus OR phizoma atractylodes macrocephala OR vitis vinifera OR melaleuca alternifolia OR lavandula angustifolia OR cupressus sempervirens OR origanum majorana OR horse chestnut OR aesculus hippocastanum OR unguentum lymphaticus OR calmodulin OR hyoscyamine OR digitalis OR cholchicine OR podophyllin OR melilotus officianalis)) OR (MH "Medicine, Herbal") OR herb therapy OR herbal therapy OR Herbalism OR Hawaiian Herbal Medicine OR "Laau Lapaau" OR "Laau Lapaau" OR "La'au Lapa'au" OR MH "Plants, Medicinal" OR Medicinal Plant OR Medicinal Plants OR Medicinal Herbs OR Medicinal Herb OR Pharmaceutical Plants OR Pharmaceutical Plant OR Healing Plants OR Healing Plant) OR MH "Dietary Supplements+" OR “Dietary Supplement” OR “Dietary Supplementations” OR “Food Supplementations” OR “Food Supplements” OR “Food Supplement” OR Nutraceuticals OR Nutraceutical OR Nutriceuticals OR Nutriceutical OR Neutraceuticals OR Neutraceutical OR “Herbal Supplements” OR “Herbal Supplement”))) AND ("Breast Cancer Lymphedema" OR Breast Cancer Lymphedemas OR Breast Cancer Related Lymphedema OR Breast Cancer Treatment-Related Lymphedema OR Breast Cancer Treatment Related Lymphedema OR Breast Cancer-Related Arm Lymphedema OR Breast Cancer Related Arm Lymphedema OR Postmastectomy Lymphedema OR Postmastectomy Lymphedemas OR Post-mastectomy Lymphedema OR Post mastectomy Lymphedema OR Post-mastectomy Lymphedemas))) | | | | |
| **2E.** Scopus search strategy | | | | |
| (“56 BaP” OR benzopyrone OR coumarin OR "cyclo 3 fort" OR “hesperidin methylchalcone” OR “ruscus aculeatus” OR “butcher's-broom” OR daflon OR diosmin OR “diosmetin 7-o-rutinoside” OR hesperidin OR "BN 165" OR “ginkgo biloba” OR troxerutin OR “foeniculum vulgare” OR fennel OR “salvia officinalis” OR sage OR geranium OR juniper OR “black pepper” OR “piper nigrum” OR oak OR “quercus robur” OR robuvit OR kampo OR goreisan OR goshajinkigan OR linfadren OR arbutin OR “weiyang huoxue washin” OR “zingiber officinale rosc” OR cinnamomum OR zanthoxylum OR “angelica sinensis” OR “sairei to” OR “chai-ling-tang” OR “bupleurum root” OR “pinellia tuber” OR “alisma rhizome” OR “scutellaria root” OR ginseng OR “poria sclerotium” OR “polyporus sclerotium” OR “atractylodes lancea rhizome” OR jujube OR glycyrrhiza OR licorice OR cinnamon OR saikosaponin OR lypmhomyosot OR “gentiana lutea” OR “pinus sylvestris” OR “scrophularia nodosa” OR “equisetum hyemale” OR “fumaria officianalis” OR “geranium robertianum” OR “nasturtium officinale” OR sarsaparilla OR “myositis arvensis” OR “teucrium scorodonia” OR “veronica officianalis” OR sulfuretin OR “rhus verniciflua stokes” OR butein OR juzentaihoto OR “tara spinosa” OR “caesalpinia spinosa” OR “achillea millefolium” OR “centella asiatica” OR olivari OR olive OR “olea europaea” OR "3,4-dihydroxyphenyl ethanol" OR hydroxytyrosol OR “wu ling san” OR “rhizoma alismatis” OR polyporus OR “phizoma atractylodes macrocephala” OR “vitis vinifera” OR “melaleuca alternifolia” OR “lavandula angustifolia” OR “cupressus sempervirens” OR “origanum majorana” OR “horse chestnut” OR “aesculus hippocastanum” OR “unguentum lymphaticus” OR calmodulin OR hyoscyamine OR digitalis OR cholchicine OR podophyllin OR “melilotus officianalis” OR “herbal medicine” OR “herb therapy” OR “herbal therapy” OR Herbalism OR “Hawaiian Herbal Medicine” OR "Laau Lapaau" OR "Laau Lapaau" OR "La'au Lapa'au" OR “Medicinal Plant” OR “Medicinal Plants” OR “Medicinal Herbs” OR “Medicinal Herb” OR “Pharmaceutical Plants” OR “Pharmaceutical Plant” OR “Healing Plants” OR “Healing Plant” OR “Dietary Supplement” OR “Dietary Supplementations” OR “Food Supplementations” OR “Food Supplements” OR “Food Supplement” OR Nutraceuticals OR Nutraceutical OR Nutriceuticals OR Nutriceutical OR Neutraceuticals OR Neutraceutical OR “Herbal Supplements” OR “Herbal Supplement” OR "Aspirin" OR “2-(Acetyloxy)benzoic Acid” OR “Acetylsalicylic Acid” OR Solprin OR Polopirin OR Solupsan OR Easprin OR Zorprin OR Acetysal OR Acylpyrin OR Colfarit OR Ecotrin OR Endosprin OR Magnecyl OR Micristin OR Polopiryna OR Aloxiprimum OR Dispril OR "Antiinflammatory Agents, Non-Steroidal" OR “Non-Steroidal Anti-Inflammatory Agent” OR “Non Steroidal Anti Inflammatory Agent” OR NSAID OR NSAIDs OR “Nonsteroidal Anti-Inflammatory Agent” OR “Nonsteroidal Anti Inflammatory Agent” OR “Nonsteroidal Antiinflammatory Agents” OR “Non-Steroidal Anti-Inflammatory Agents” OR “Non Steroidal Anti Inflammatory Agents” OR “Nonsteroidal Anti-Inflammatory Agents” OR “Nonsteroidal Anti Inflammatory Agents” OR “Anti-Inflammatory Analgesics” OR “Aspirin-Like Agents” OR “Aspirin Like Agents” OR “Aspirin-Like Agent” OR “Aspirin Like Agent OR "Ketoprofen" OR "2-(3-Benzoylphenyl)propionic Acid” OR “Benzoylhydratropic Acid" OR Alrheumum OR Orudis OR Profenid OR "19,583 RP" OR "RP, 19,583" OR "RP-19583" OR "RP19583" OR "RP 19583" OR Alrheumat OR ibuprofen OR "alpha-Methyl-4-(2-methylpropyl)benzeneacetic Acid” OR “Benzeneacetic Acid, alpha-methyl-4-(2-methylpropyl)- trimethylsilyl ester" OR “Ibuprofen-Zinc” OR “Ibuprofen Zinc” OR Salprofen OR Brufen OR Motrin OR Ibumetin OR Nuprin OR Rufen OR “Trauma-Dolgit Gel” OR “Trauma Dolgit Gel” OR Advil OR “Anticoagulant Drug” OR “Anticoagulant Agent” OR “Anticoagulation Agents” OR “Anticoagulant Drugs” OR “Anticoagulant Agents” OR Anticoagulant OR “Indirect Thrombin Inhibitors” OR “apixaban” OR apixaban OR Eliquis OR "BMS 562247" OR "BMS-562247" OR "BMS562247" OR "BMS-562247-01" OR "Rivaroxaban” OR "BAY 59 7939" OR Xarelto OR "5-chloro-N-(((5S)-2-oxo-3-(4-(3-oxomorpholin-4-yl)phenyl)-1,3-oxazolidin-5-yl)methyl)thiophene-2-carboxamide" OR "BAY 59-7939" OR "BAY 597939" OR Edoxaban OR “edoxaban” OR "N-(5-chloropyridin-2-yl)-N'-((1S,2R,4S)-4-(N,N-dimethylcarbamoyl)-2-(5-methyl-4,5,6,7- tetrahydro(1,3)thiazolo(5,4-c)pyridine-2-carboxamido)cyclohexyl)oxamide" OR "DU-176b" OR "DU-176" OR “edoxaban tosylate” OR "N-(5-chloropyridin-2-yl)-N'-((1S,2R,4S)-4-(N,N-dimethylcarbamoyl)-2-(5-methyl-4,5,6,7-tetrahydrothiazolo(5,4-c)pyridine-2-carboxamido)cyclohexyl)ethanediamide p-toluenesulfonate monohydrate" OR Savaysa OR “Fondaparinux” OR Fondaparinux OR “Fondaparinux Sodium” OR Arixtra OR Quixidar OR Heparin OR “Low Molecular Weight Heparin” OR “Low-Molecular Weight Heparin” OR LMWH OR “Heparinic Acid” OR “Unfractionated Heparin” OR “Sodium Heparin” OR “Heparin Sodium” OR “alpha-Heparin” OR “alpha Heparin” OR Liquaemin OR Dalteparin OR Tedelparin OR “Dalteparin Sodium” OR Fragmin OR Fragmine OR "Kabi-2165" OR "Kabi2165" OR "Kabi 2165" OR "FR-860" OR "FR860" OR "FR 860" OR Enoxaparin OR Enoxaparine OR "EMT-966" OR "EMT966" OR "EMT 966" OR "EMT-967" OR "EMT967" OR "EMT 967" OR Clexane OR "PK-10,169" OR "PK 10,169" OR "PK10,169" OR "PK-10169" OR "PK10169" OR "PK 10169" OR Lovenox OR Argatroban OR "2R,4R)-1-(5-((aminoiminomethyl)amino)-1-oxo-2-(((1,2,3,4-tetrahydro-3-methyl-8-quinolinyl)sulfonyl)amino)pentyl)-4-methyl-2-piperidinecarboxylic acid monohydrate” OR MMTQAP OR MPQA OR “argatroban monohydrate” OR “argatroban hydrate” OR "MD 805" OR "MD-805" OR "MD805" OR Acova OR Novastan OR “argatroban anhydrous” OR "(2R,4R)-1-(5-((aminoiminomethyl)amino)-1-oxo-2-(((1,2,3,4-tetrahydro-3-methyl-8-quinolinyl)sulfonyl)amino)pentyl)-4-methyl-2-piperidinecarboxylic acid" OR "(21R)-argatroban anhydrous" OR "(2R,4R)-1-((2S)-5-((aminoiminomethyl)amino)-1-oxo-2-((((3R)-1,2,3,4-tetrahydro-3-methyl-8-quinolinyl)sulfonyl)amino)pentyl)-4-methyl-2-piperidinecarboxylic acid" OR "(21S)-argatroban anhydrous PR (2R,4R)-1-((2S)-5-((aminoiminomethyl)amino)-1-oxo-2-((((3S)-1,2,3,4-tetrahydro-3-methyl-8-quinolinyl)sulfonyl)amino)pentyl)-4-methyl-2-piperidinecarboxylic acid" OR "(21S)-argatroban" OR "MCI 9038" OR "MCI-9038" OR “(21R)-argatroban)” OR bivalirudin OR "L-leucine, D-phenylalanyl-L-prolyl-L-arginyl-L-prolylglycylglycylglycylglycyl-L-asparaginylglycyl-L-alpha-aspartyl-L-phenylalanyl-L-alpha-glutamyl-L-alpha-glutamyl-L-isoleucyl-L-prolyl-L-alpha-glutamyl-L-alpha-glutamyl-L-tyrosyl- OR Phe-Pro-Arg-Pro-(Gly)4 desulfato-Tyr63'-hirugen" OR "Phe-Pro-Arg-Pro-(Gly)4-Asn-Gly-Asp-Phe-Glu-Glu-Ile-Pro-Glu-Glu-Tyr-Leu OR Phe-Pro-Arg-Pro-(Gly)4-desulfohirudin-(53-64)" OR "BG 8967" OR "BG-8967" OR "BG8967" OR Hirulog OR “Hirulog-1” OR Angiomax OR “Angiomax RTU” OR "CTB-001" OR bivalirudin trifluoroacetate” OR Dabigatran OR "N-((2-(((4-(aminoiminomethyl)phenyl)amino)methyl)-1-methyl-1H-benzimidazol-5-yl)carbonyl)-N-2-pyridinyl-beta-alanine" OR “Dabigatran Etexilate” OR "BIBR 1048" OR Pradaxa OR “Dabigatran Etexilate Mesylate” OR Desirudin OR desulfatohirudin OR desulphatohirudin OR “recombinant HV1 hirudin” OR "CGP 39393" OR "CGP-39393" OR "IK-HIR02" OR Revasc OR Iprivask OR Warfarin OR Coumadin OR "4-Hydroxy-3-(3-oxo-1-phenylbutyl)-2H-1-benzopyran-2-one" OR “Warfarin Potassium” OR “Warfarin Sodium” OR Marevan OR Gen-Warfarin OR Aldocumar OR Coumadine OR Apo-Warfarin OR Tedicumar OR Warfant OR Cyclosporin OR Ciclosporin OR Cyclosporin OR “Cyclosporin A” OR “Cyclosporine A” OR “CsA-Neoral” OR CsANeoral OR “CsA Neoral” OR “Sandimmun Neoral” OR CyA-NOF OR CyA NOF OR Sandimmune OR Sandimmun OR Neoral OR "OL 27-400" OR "OL 27400" OR "OL 27 400" OR hydroxychloroquine OR Hydroxychlorochin OR Oxychloroquine OR Oxychlorochin OR Plaquenil OR “Hydroxychloroquine Sulfate” OR “Hydroxychloroquine Sulfate (1:1) Salt” OR Tacrolimus OR Prograft OR :Anhydrous Tacrolimus” OR "FK-506" OR "FK 506" OR "FK506" OR "FR-900506" OR "FR900506" OR "FR 900506" OR Sirolimus OR Rapamycin OR Rapamune OR "AY 22-989" OR "AY 22989" OR "AY 22 989" OR "I-2190A" OR "I2190A" OR "I 2190A" OR “Leukotriene B4 antagonist” OR “Leukotriene B4 inhibitor” OR “Leukotriene B4” OR “Leukotriene B-4” OR "Leukotriene B 4" OR LTB4 OR "5,12-diHETE" OR "5,12 diHETE" OR "5,12-HETE" OR "5,12 HETE" OR "Leukotrienes B" OR "Leukotriene B" OR  “Platelet Rich Plasma” OR “Platelet-Rich Plasma” OR “Immunosuppressive Agent” OR Immunosuppressant OR Immunosuppressants AND "Breast Cancer Lymphedema" OR “Breast Cancer Lymphedemas” OR “Breast Cancer Related Lymphedema” OR “Breast Cancer Treatment-Related Lymphedema” OR “Breast Cancer Treatment-Related Lymphedemas” OR “Breast Cancer Treatment Related Lymphedema” OR “Breast Cancer Treatment Related Lymphedemas” OR “Breast Cancer-Related Arm Lymphedema” OR “Breast Cancer-Related Arm Lymphedemas” OR “Breast Cancer Related Arm Lymphedema” OR “Breast Cancer Related Arm Lymphedemas” OR “Postmastectomy Lymphedema” OR “Postmastectomy Lymphedemas” OR “Post-mastectomy Lymphedema” OR “Post mastectomy Lymphedema” OR “Post-mastectomy Lymphedemas” | | | | |
| **2F.** Web of Science search strategy | | | | |
| (TS=(56 BaP OR benzopyrone OR coumarin OR “cyclo 3 fort" OR "hesperidin methylchalcone" OR "ruscus aculeatus" OR "butcher's-broom" OR daflon OR diosmin OR "diosmetin 7-o-rutinoside" OR hesperidin OR "BN 165" OR "ginkgo biloba" OR troxerutin OR "foeniculum vulgare" OR fennel OR "salvia officinalis" OR sage OR geranium OR juniper) OR TS=(glycyrrhiza OR licorice OR cinnamon OR saikosaponin OR lymphomyosot OR "gentiana lutea" OR "pinus sylvestris" OR "scrophularia nodosa" OR "equisetum hyemale" OR "fumaria officianalis" OR "geranium robertianum" OR "nasturtium officinale" OR sarsaparilla OR "myositis arvensis" OR "teucrium scorodonia" OR "veronica officianalis" OR sulfuretin OR "rhus verniciflua stokes" ) OR TS=(butein OR juzentaihoto OR "tara spinosa" OR "caesalpinia spinosa" OR "achillea millefolium" OR "centella asiatica" OR olivary OR olive OR "olea europaea" OR "3,4-dihydroxyphenyl ethanol" OR hydroxytyrosol OR "wu ling san" OR "rhizoma alismatis" OR polyporus OR "phizoma atractylodes macrocephala" OR "vitis vinifera" ) OR TS=("melaleuca alternifolia" OR "lavandula angustifolia" OR "cupressus sempervirens" OR "origanum majorana" OR "horse chestnut" OR "aesculus hippocastanum" OR "unguentum lymphaticus" OR calmodulin OR hyoscyamine OR digitalis OR cholchicine OR podophyllin OR "melilotus officianalis" OR "Phytotherapy" OR "herb therapy" OR "herbal therapy") OR TS=("Herbal Medicine" OR Herbalism OR "Hawaiian Herbal Medicine" OR "Laau Lapaau" OR "La'au Lapa'au" OR "Plants, Medicinal" OR "Medicinal Plant" OR "Medicinal Plants" OR "Medicinal Herbs" OR "Medicinal Herb" OR "Pharmaceutical Plants" OR "Pharmaceutical Plant" OR "Healing Plants" OR "Healing Plant" OR "Dietary Supplements" OR "Dietary Supplement" OR "Dietary Supplementations") OR TS=( "Food Supplementations" OR "Food Supplements" OR "Food Supplement" OR Nutraceuticals OR Nutriceuticals OR Nutriceutical OR Neutraceuticals OR “Herbal Supplements” OR "Herbal Supplement" ) OR TS=(Aspirin OR “2-(Acetyloxy)benzoic Acid” OR “Acetylsalicylic Acid” OR solarin OR polopirin OR soluspan OR easpirin OR zaiprin OR acetosal OR acylpyran OR collaris OR ecotron OR endosperin OR magnesyl OR mycristin OR polopirin OR aloxiprinum OR disprin OR “Anti-Inflammatory Agents, Non-Steroidal” OR “Non-Steroidal Anti-Inflammatory Agent” OR “Non Steroidal Anti Inflammatory Agent” OR NSAID OR NSAIDs OR “Nonsteroidal Anti-Inflammatory Agent” OR “Nonsteroidal Anti Inflammatory Agent” OR “Nonsteroidal Antiinflammatory Agents” OR “Non-Steroidal Anti-Inflammatory Agents” OR “Non Steroidal Anti Inflammatory Agents” OR “Nonsteroidal Anti-Inflammatory Agents” OR “Nonsteroidal Anti Inflammatory Agents” OR “Anti-Inflammatory Analgesics” OR “Aspirin-Like Agents” OR “Aspirin Like Agents” OR “Aspirin-Like Agent” OR “Aspirin Like Agent” ) OR TS=(Ketoprofen OR "2-(3-Benzoylphenyl)propionic Acid OR Benzoylhydratropic Acid" OR alrheumun OR ordis OR profenic OR "19,583 RP" OR "RP, 19,583" OR "RP-19583" OR "rs17583" OR "RP 19583" OR alrheumun OR Ibuprofen OR "alpha-Methyl-4-(2-methylpropyl)benzeneacetic Acid OR Benzeneacetic Acid, alpha-methyl-4-(2-methylpropyl)- trimethylsilyl ester" OR “Ibuprofen-Zinc” OR “Ibuprofen Zinc” OR salpropen OR brumen OR matrin OR ibumetinr OR nuprid OR rumen OR “Trauma-Dolgit Gel” OR “Trauma Dolgit Gel” OR anvil ) OR TS=(Anticoagulants OR “Anticoagulant Drug” OR “Anticoagulant Agent” OR “Anticoagulation Agents” OR “Anticoagulant Drugs” OR “Anticoagulant Agents” OR Anticoagulant OR “Indirect Thrombin Inhibitors” OR apixaban OR eliquids OR "BMS 562247" OR "BMS-562247" OR "BMS562247" OR "BMS-562247-01" OR Rivaroxaban OR "BAY 59 7939" OR Xarelto OR "5-chloro-N-(((5S)-2-oxo-3-(4-(3-oxomorpholin-4-yl)phenyl)-1,3-oxazolidin-5-yl)methyl)thiophene-2-carboxamide" OR "BAY 59-7939" OR "BAY 597939") OR TS=(Edoxaban OR "N-(5-chloropyridin-2-yl)-N'-((1S,2R,4S)-4-(N,N-dimethylcarbamoyl)-2-(5-methyl-4,5,6,7- tetrahydro(1,3)thiazolo(5,4-c)pyridine-2-carboxamido)cyclohexyl)oxamide" OR "DU-176b" OR "DU-176" OR “edoxaban tosylate” OR "N-(5-chloropyridin-2-yl)-N'-((1S,2R,4S)-4-(N,N-dimethylcarbamoyl)-2-(5-methyl-4,5,6,7-tetrahydrothiazolo(5,4-c)pyridine-2-carboxamido)cyclohexyl)ethanediamide p-toluenesulfonate monohydrate" OR savasa OR Fondaparinux OR “Fondaparinux Sodium” OR Arixtra OR quixada OR Heparin OR “Low Molecular Weight Heparin” OR LMWH OR “Heparinic Acid” OR “Unfractionated Heparin” OR “Sodium Heparin” OR “Heparin Sodium” OR “alpha-Heparin” OR “alpha Heparin” OR liquemin OR Dalteparin OR Dalteparin OR Tedelparin OR “Dalteparin Sodium” OR Fragmin OR fragminr OR "Kabi-2165" OR "Kabi2165" OR "Kabi 2165" OR "FR-860" OR "fr160" OR "FR 860" OR Enoxaparin OR Enoxaparine OR "EMT-966" OR "emtp96" OR "EMT 966" OR "EMT-967" OR "emtp96" OR "EMT 967" OR Clexane OR "PK-10,169" OR "PK 10,169" OR "PK10,169" OR "PK-10169" OR "pp10164" OR "PK 10169" OR Lovenox ) OR TS=(Argatroban OR "2R,4R)-1-(5-((aminoiminomethyl)amino)-1-oxo-2-(((1,2,3,4-tetrahydro-3-methyl-8-quinolinyl)sulfonyl)amino)pentyl)-4-methyl-2-piperidinecarboxylic acid monohydrate OR MMTQAP OR MPQA" OR “argatroban monohydrate” OR “argatroban hydrate” OR "MD 805" OR "MD-805" OR "md800" OR anova OR novascan OR “argatroban anhydrous” OR "(2R,4R)-1-((2S)-5-((aminoiminomethyl)amino)-1-oxo-2-((((3R)-1,2,3,4-tetrahydro-3-methyl-8-quinolinyl)sulfonyl)amino)pentyl)-4-methyl-2-piperidinecarboxylic acid" OR "(21R)-argatroban anhydrous" OR "(2R,4R)-1-((2S)-5-((aminoiminomethyl)amino)-1-oxo-2-((((3S)-1,2,3,4-tetrahydro-3-methyl-8-quinolinyl)sulfonyl)amino)pentyl)-4-methyl-2-piperidinecarboxylic acid" OR "(21S)-argatroban anhydrous PR (2R,4R)-1-((2S)-5-((aminoiminomethyl)amino)-1-oxo-2-((((3S)-1,2,3,4-tetrahydro-3-methyl-8-quinolinyl)sulfonyl)amino)pentyl)-4-methyl-2-piperidinecarboxylic acid" OR "(21S)-argatroban" OR "MCI 9038" OR "MCI-9038" OR “(21R)-argatroban OR bivalirudin” OR "L-leucine, D-phenylalanyl-L-prolyl-L-arginyl-L-prolylglycylglycylglycylglycyl-L-asparaginylglycyl-L-alpha-aspartyl-L-phenylalanyl-L-alpha-glutamyl-L-alpha-glutamyl-L-isoleucyl-L-prolyl-L-alpha-glutamyl-L-alpha-glutamyl-L-tyrosyl-OR Phe-Pro-Arg-Pro-(Gly)4 desulfato-Tyr63'-hirugen" OR "Phe-Pro-Arg-Pro-(Gly)4-Asn-Gly-Asp-Phe-Glu-Glu-Ile-Pro-Glu-Glu-Tyr-Leu” OR “Phe-Pro-Arg-Pro-(Gly)4-desulfohirudin-(53-64)" OR "BG 8967" OR "BG-8967" OR "b28767" OR Hirulog OR Hirulog-1 OR angiomas OR “angiomas RTU” OR "CTB-001" OR “bivalirudin trifluoroacetate” ) OR TS=(Dabigatran OR "N-((2-(((4-(aminoiminomethyl)phenyl)amino)methyl)-1-methyl-1H-benzimidazol-5-yl)carbonyl)-N-2-pyridinyl-beta-alanine" OR “Dabigatran Etexilate” OR "BIBR 1048" OR Pradaxa OR “Dabigatran Etexilate Mesylate” OR Desirudin OR desulfatohirudin OR desulfatohirudin OR “recombinant HV1 hirudin” OR "CGP 39393" OR "CGP-39393" OR "IK-HIR02" OR revascu OR irivasc OR Warfarin OR Warfarin OR Coumadin OR "4-Hydroxy-3-(3-oxo-1-phenylbutyl)-2H-1-benzopyran-2-one" OR “Warfarin Potassium” OR “Warfarin Sodium” OR marivan OR “Gen-Warfarin” OR andocular OR coumarine OR “Apo-Warfarin” OR tesicular OR warman) OR TS=(Cyclosporine OR Cyclosporin OR Ciclosporin OR Cyclosporin OR “Cyclosporin A” OR “CsA-Neoral” OR cantoral OR “CsA Neoral” OR “Sandimmun Neoral” OR “CyA-NOF” OR “CyA NOF” OR Sandimmune OR Sandimmun OR Neoral OR "OL 27-400" OR "OL 27400" OR "OL 27 400" OR Hydroxychloroquine OR hydroxychlorochine OR ofchloroquine OR oxychloraphin OR Plaquenil OR “Hydroxychloroquine Sulfate” OR “Hydroxychloroquine Sulfate (1:1) Salt” OR Tacrolimus OR pregraft OR “Anhydrous Tacrolimus” OR "FK-506" OR "FK 506" OR "FK506" OR "FR-900506" OR "fr000506" OR "FR 900506" OR Sirolimus OR Rapamycin OR Rapamune OR "AY 22-989" OR "AY 22989" OR "AY 22 989" OR "I-2190A" OR "i2190n" OR "I 2190A" ) OR TS=(“Leukotriene B4 antagonist” OR “Leukotriene B4 inhibitor” OR “Leukotriene B4” OR “Leukotriene B-4” OR "Leukotriene B 4" OR LTB4 OR "5,12-diHETE" OR "5,12 diHETE" OR "5,12-HETE" OR "5,12 HETE" OR "Leukotrienes B" OR "Leukotriene B" OR “Platelet-Rich Plasma” OR “Platelet Rich Plasma” OR “Platelet-Rich Plasma” OR “Immunosuppressive Agents” OR “Immunosuppressive Agent” OR Immunosuppressant OR Immunosuppressants )) AND TS=(“Breast Cancer Lymphedema” OR “Breast Cancer Lymphedemas” OR “Breast Cancer Related Lymphedema” OR “Breast Cancer Treatment-Related Lymphedema” OR “Breast Cancer Treatment Related Lymphedema” OR “Breast Cancer-Related Arm Lymphedema” OR “Breast Cancer Related Arm Lymphedema” OR “Postmastectomy Lymphedema” OR “Postmastectomy Lymphedemas” OR “Post-mastectomy Lymphedema” OR “Post mastectomy Lymphedema” OR “Post-mastectomy Lymphedemas”) | | | | |
